# The genus *Mercuria* Boeters, 1971 in Morocco: first molecular phylogeny of the genus and description of two new species (Caenogastropoda, Truncatelloidea, Hydrobiidae)

**DOI:** 10.3897/zookeys.782.26797

**Published:** 2018-08-27

**Authors:** Khadija Boulaassafer, Mohamed Ghamizi, Diana Delicado

**Affiliations:** 1 Cadi Ayyad University, Faculty of Science, Department of Biology, Hydrobiology, Ecotoxicology, Sanitation and Climate Change, Prince Moulay Abdellah Boulevard, Marrakesh 40000, Morocco Cadi Ayyad University Marrakesh Morocco; 2 Justus Liebig University, Department of Animal Ecology & Systematics, Heinrich-Buff-Ring 26-32, D-35392, Giessen, Germany Justus Liebig University Giessen Germany

**Keywords:** Anatomy, endemism, freshwater, molluscs, mtCOI, parasitism, systematics

## Abstract

The western Palearctic freshwater snail genus *Mercuria* (Caenogastropoda: Hydrobiidae) comprises 26 species primarily distributed in lowland localities of Western Europe and North Africa. Although this genus in North Africa has received considerable attention in terms of species discoveries through morphological descriptions, its distribution and phylogenetic patterns remain unknown. Based on morphological and mitochondrial DNA (mtCOI) evidence, this study examines the three *Mercuria* species (*M.bakeri*, *M.tingitana*, and *M.targouasensis*) from Morocco identified so far. Besides expanding on information regarding the anatomy of these species, two new species (*M.midarensis***sp. n.** and *M.tensiftensis***sp. n.**) are described for this region and phylogenetic relationships inferred between these species and the European *M.emiliana* and *M.similis*. All Moroccan and European species were recovered as independent entities according to these phylogenetic inferences (uncorrected p-distances 2.8–8.5%) and DNA barcode data. Moroccan *Mercuria* species clustered with *M.emiliana* from Spain, although basal relationships within this clade were not well supported. Given that factors such as the season when specimens are collected, habitat type, and parasites could be responsible for the remarkable intraspecific variation observed in shell and penis morphology, it is proposed that the most efficient approach to delimit and identify *Mercuria* species is to combine morphological descriptions with genetic data.

## Introduction

The gastropod genus *Mercuria* Boeters, 1971 (Truncatelloidea, Hydrobiidae) is widely distributed in continental aquatic systems of western Mediterranean territories and islands (Giusti 1979, Boeters 1988, Glöer et al. 2010, Patzner and Glöer 2013, Boeters and Falkner 2017) and more rarely in those of the Atlantic coasts of North Africa (Glöer et al. 2015) and Western Europe (Boeters 1988, Kadolsky 2011, Glöer et al. 2015, Boeters and Falkner 2017) and Madeira (Glöer et al. 2015). This genus currently comprises 26 species occurring mainly in lowland regions of North Africa (nine species; Glöer et al. 2015), continental France (six species; Boeters and Falkner 2017), and the Iberian Peninsula and Balearic Islands (four species, Boeters 1988, Glöer et al. 2015). *Mercuria* species occupy a wide variety of aquatic habitats, typically living in high abundance in springs and their outflows, coastal streams, and tide areas of rivers. Less frequently they appear in brackish meadows and ponds. Despite their high representation in lowland aquatic biotopes, phylogenetic relationships among *Mercuria* congeners have been scarcely explored. Species assignments of these snails have been based on conchological and anatomical studies. However, given their small size (shell height 1–5 mm) and featureless shells, molecular tools could help confirm the taxonomy and the phylogenetic position of *Mercuria* within the family. Based on molecular data obtained for the species *Mercuriasimilis* (Draparnaud, 1805), Wilke et al. (2013) recovered this genus as an independent lineage within the Hydrobiidae, which was further designated as the subfamily Mercuriinae (Boeters and Falkner 2017).

Northwestern Morocco harbors a relatively large proportion (four species) of the *Mercuria* species richness of North Africa. The earliest record of this genus in southern Morocco was *M.confusa* (Frauenfeld, 1863) (BackHuys and Boeters 1974), which has been recently placed in synonymy with *M.similis* (Boeters and Falkner 2017). Over the years, *M.similis* (=*M.confusa*) has been reported from several springs and streams in central and western Morocco (Ghamizi et al. 1997, Berrahou et al. 2001, Touabay et al. 2002, Boulal et al. 2017, Taybi et al. 2017). However, these records require confirmation through additional anatomical descriptions and molecular data. Based on penis and shell features, Glöer et al. (2015) recently described three new species, each found only in a single pond or spring in the coastal regions of Morocco: *M.tingitana*, *M.bakeri*, and *M.targouasensis*. Moreover, these authors identified two potential new *Mercuria* species but these have not been formally described. One was previously referred to as *M.* ‘*mirlheftensis*’ (nomen nudum) by García et al. (2010), who included this species as ‘Endangered’ in the IUCN Red List of Threatened Species. However, this taxon has not been properly described and this name cannot therefore be considered valid.

Here we examined morphologically, anatomically, and molecularly a few paratypes of the species *M.tingitana* and *M.bakeri* as well as individuals from the population of *M.* ‘*mirlheftensis*’ and other Moroccan localities. Our objectives were: (1) to delimit the formerly known and potential new Moroccan species of *Mercuria* under the phylogenetic species concept (i.e., a monophyletic assemblage of populations that possesses a unique combination of morphological traits) previously applied to hydrobiids (e.g., Delicado et al. 2012, Hershler et al. 2013); (2) to examine intra- and interspecific genetic variation and resolve phylogenetic relationships based on mtCOI sequences; and (3), to provide habitat and ecological data for future conservation plans.

## Materials and methods

**Figure 1.**
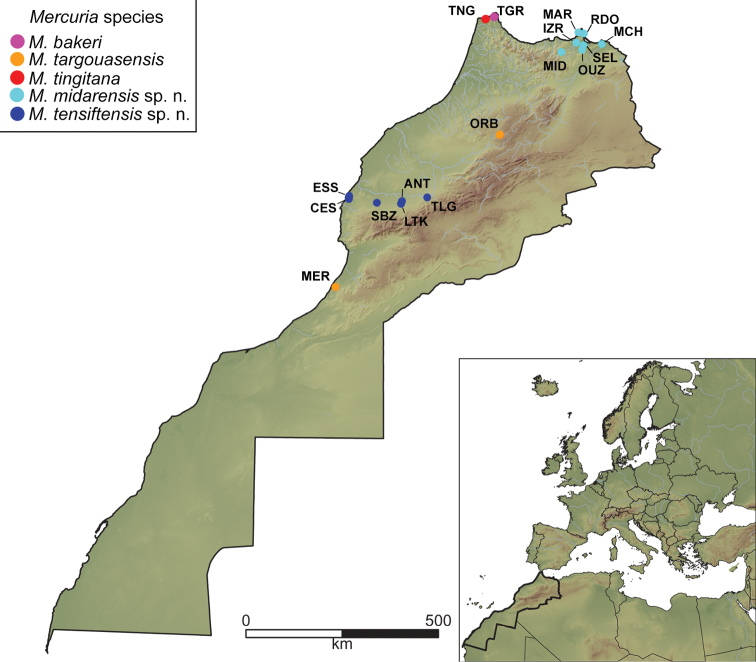
Map of Morocco showing sample locations and codes of the studied *Mercuria* populations. Locality codes are from Table 1.

Individuals of *Mercuria* were collected from 16 localities, ranging from north-eastern to south-western Morocco (Figure 1), either by sieving mud or by hand with fine forceps. For anatomical studies, a share of the specimens of every locality was relaxed with menthol crystals following the protocols described in Ramos et al. (2000) and Arconada and Ramos (2001) and then preserved in 80% ethanol. The remaining specimens were preserved directly in 80% ethanol for genetic analyses. Type material was deposited into the Museo Nacional de Ciencias Naturales (**MNCN**) collection, Madrid, Spain. Voucher material and DNA samples were deposited in the University of Giessen Systematics and Biodiversity (**UGSB**) collection (Diehl et al. 2018) in Germany and in the Hydrobiid collection at Muséum d’Histoire Naturelle de Marrakech in Morocco.

We sequenced two *Mercuria* specimens per locality in most cases and analyzed together with other *Mercuria* sequences available in GenBank (Table 1). The final dataset comprised 30 sequences and two outgroup taxa, *Pseudamnicolalucensis* (Issel, 1866) and *Pyrgulopsisbedfordensis* (Hershler & Gustafson, 2001) [GenBank accession numbers AF367651 (Wilke et al. 2001) and EU700483 (Hershler et al. 2008), respectively]. Genomic DNA was extracted following the CTAB protocol of Wilke et al. (2006). LCO1490 (Folmer et al. 1994) and COR722b (Wilke and Davis 2000) primers were used to amplify a fragment of the cytochrome *c* oxidase subunit I (COI) gene. A shorter fragment of COI was obtained for *M.bakeri* and *M.tingitana* by using the sequence PCOIF4 (5’-TAGCTATTTTTTCTYTACATYTRGC-3’) as forward and COR722b as reverse primers. The PCR cycling conditions were as described in Schreiber et al. (2012) and Delicado et al. (2012). Products were sequenced in an ABI 3730 XL sequencer (Life Technologies, Carlsbad, CA, USA) using a Big Dye Terminator Kit (Life Technologies). New DNA sequences were edited in SEQUENCHER 4.6 (Gene Codes, Ann Arbor, MI), assembled together with sequences from GenBank and unambiguously aligned using MEGA 7.0. (Kumar et al. 2016). Sequence divergences (uncorrected p-distances) were also calculated in MEGA. Prior to the phylogenetic analyses, we employed jModelTest 2.1.4 (Darriba et al. 2012) under corrected Akaike’s information criterion (AICc; Akaike 1974, Sugiura 1978, Hurvich and Tsai 1989) to obtain the best-fit substitution model for our data set.

Phylogenetic relationships among European and Moroccan *Mercuria* species were inferred based on Maximum Likelihood (ML) and Bayesian Inference (BI) using the substitution model HKY (Hasegawa et al. 1985) +G (including variation among sites) suggested by jModelTest. ML analysis was conducted in PhyML v. 3.1 (Guindon et al. 2010) with the above-mentioned evolutionary model and 100 random starting trees. Bayesian inference was performed with MrBayes 3.2.1 (Huelsenbeck and Ronquist 2001, Ronquist and Huelsenbeck 2003) through two independent runs of four parallel Markov Chain Monte Carlo (MCMC) simulations with 1.5 million generations each and a sample interval of 1000 generations. Convergence of the MCMC chains was examined by ensuring an average standard deviation of split frequencies lower than 0.01 in MrBayes and by checking with Tracer 1.6 (Rambaut et al. 2014) that all effective sample sizes (ESS) were higher than 200. After discarding the first 10% of the trees (burn-in), the remaining trees were used to construct a 50% majority-rule-consensus tree. Branch support was assessed by nonparametric bootstrapping (Felsenstein 1985) using 1000 pseudoreplicates for ML and by posterior probabilities (BPPs) for the BI. Trees and support values of branches were finally visualized in FigTree 1.3.1 (Rambaut 2010).

Additionally, we tested the assignment of the Moroccan sequences to the species identified as new by the ML and BI approaches using the Automatic Barcode Gap Discovery (ABGD: Puillandre et al. 2012). The ABGD analysis was conducted at the web interface http://wwwabi.snv.jussieu.fr/public/abgd/ using the aligned matrix of COI sequences as input file, and the default settings, which consisted in the uncorrected genetic distances, a relative gap width of X = 1.5, and intraspecific divergence (P) values between 0.001 and 0.100.

A series of adults (number specified in the corresponding sections of the text and tables) from each locality and a few paratypes of the species *M.tingitana* and *M.bakeri* were morphologically examined. Morphological and anatomical characteristics were studied under a binocular Olympus SZX12 and photographed using a Keyence VHX 2000 3D Digital Microscope in combination with the program VHX-2000 Communication software version 2.3.5.0 (Keyence Corporation 2009–2012). Spire whorls were counted following Ramos et al. (2000). Before dissection, shells were dissolved in HCl or Ethylenediaminetetraacetic acid (EDTA). Radulae were extracted by applying the first step of the Proteinase K protocol for DNA isolation (Wilke et al. 2006). After mounting on stubs and drying, radulae were covered with gold (Balter Sputter Coater SCD004) for 50 sec. and then photographed with a field emission scanning electron microscope (FESEM) DSM982 Gemini (Carl Zeiss GmbH, Germany). Anatomical illustrations were obtained from camera lucida drawings carried out under a WILD HEERBRUGG stereomicroscope. Morphological and anatomical character states are based on the terminology of Hershler and Ponder (1998).

**Table 1.** Species name, locality information, locality code used in the phylogenetic analyses, and GenBank accession numbers for the *Mercuria*mtCOI sequences.

Species name	Locality	Locality code	GenBank number
* Mercuria similis *	Canale Panigai near Panigai, Friuli-Venetia-Julia, Udine, Aquileia, Italy (45°44.49'N, 13°20.448'E)		AF367646 (Wilke et al. 2001)
* M. emiliana *	Ullal Baltasar, Amposta, Tarragona, Spain (40°40.252'N, 00°35.212'E)		JX081888 (Delicado et al. 2013)
	Mallorca, La Puebla, Spain (39°47.467'N, 3°6.283'E)		AF213346 (Wilke et al. 2000)
* M. tingitana *	Swampy area between Tangier and Ksar es Seghir, Morocco (35°49.008'N, 5°43.644'W)	TNG	MH315899 (Present study)
* M. bakeri *	Spring at 3.5 km N of Taghramt, Morocco (35°48.912'N, 5°27.618'W)	TGR	MH315900 (Present study)
* M. targouasensis *	Ditch in Mirleft, Morocco (29°35.0167'N, 10°1.845'W)	MER	MH315885 (Present study)
	Oum Rbii Springs, Khenifra, Morocco (33°3.2059'N, 5°24.8797'W)	ORB	MH315886, MH315887 (Present study)
*M.tensiftensis* sp. n.	Ditch in Talkount, N-E of Marrakesh, Morocco (31°40.5775'N, 7°16.0298'W)	TLG	MH315888, MH315889 (Present study)
	Ditch in Sidi Bouzid, near Chichaoua, Morocco (type locality) (31°29.6133'N, 8°47.1116'W)	SBZ	MH315890, MH315891 (Present study)
	Agadir N’tachraft, S of Marrakesh, Morocco (31°23.0917'N, 8°7.5033'W)	ANT	MH315892 (Present study)
	Spring near Lalla Takerkoust Dam, Morocco (31°22.5491'N, 8°7.6385'W)	LTK	MH315893, MH315894 (Present study)
	Ditch in Haddada Bouzerktoun, Essaouira, Morocco (31°37.95'N, 9°35.0983'W)	CES	MH315895, MH315896 (Present study)
	Pond near Lahjar Spring, Essaouira, Morocco (31°38.7583'N, 9°35.0983'W)	ESS	MH315897, MH315898 (Present study)
*M.midarensis* sp. n.	Mariouari River, near Melilla, Morocco (35°18.36'N, 2°58.6483'W)	MAR	MH315874, MH315875 (Present study)
	Rio de Oro, Melilla, Spain (35°17.2483'N, 2°56.6283'W).	RDO	MH315876 (Present study)
	Izerouan River, W of Nador, Morocco (35°9.8333'N, 3°6.6'W)	IZR	MH315877 (Present study)
	Selouan River, S of Nador, Morocco (35°4.6117'N, 2°55.485'W)	SEL	MH315878, MH315879 (Present study)
	Ouzej River, Al Aaroui, Morocco (35°0.3634'N, 2°59.5133'W)	OUZ	MH315880, MH315881 (Present study)
	Ditch near Midar, Morocco (type locality) (34°54.5795'N, 3°34.0292'W)	MID	MH315882, MH315883 (Present study)
	Cherarba Ponds, W of Saidia, Morocco (35°6.3116'N, 2°20.75'W)	MCH	MH315884 (Present study)

### Abbreviations used in the text and tables

*Shell and operculum characters*: AH: aperture height; AL: aperture length; AW: aperture width; LBW: length of body whorl; NSW: number of spire whorls; SL: shell length; SW: shell width; WAW: width of the antepenultimate whorl; WBW: width of the body whorl; WPW: width of the penultimate whorl.

*Anatomical characters*: Ag: albumen gland; Bc: bursa copulatrix; CC: cerebral commissure; Cg: capsule gland; Ct: ctenidium; dBc: duct of the bursa copulatrix; LCG: left cerebral ganglion; LPG: left pleural ganglion; Os: osphradium; P: penis; PA: penial appendix; Po: pallial oviduct; Pr: prostate gland; RCG: right cerebral ganglion; Ro: renal oviduct; RPG: right pleural ganglion; SR: seminal receptacle; Ss: style sac; St: stomach; SubC: suboesophageal connective; SubG: suboesophageal ganglion; SupC: supraoesophageal connective; SupG: supraoesophageal ganglion; L: length; W: width. Concentration of the nervous system was measured as the “RPG” ratio (Davis et al. 1976) and described by applying the categories of Davis et al. (1984, 1986, 1992) as follows: dorsal nerve ring concentrated (RPG ratio ≤ 0.29); moderately concentrated (RPG ratio 0.30–0.49); elongated (RPG ratio 0.50–0.67); extremely elongated (RPG ratio ≥ 0.68).

*Collections*: MHNM: Muséum d’Histoire Naturelle de Marrakech, Morocco; MNCN: Museo Nacional de Ciencias Naturales, Madrid, Spain; UGSB: University of Giessen Systematics and Biodiversity Collection, Giessen, Germany.

*Collectors*: K.B., K. Boulaassafer, D.D., D. Delicado, M.G., M. Ghamizi, T.H., T. Hauffe.

## Results

### Phylogenetic relationships and genetic distances

The resulting COI data set yielded 658 bp. All new sequences obtained in this work were deposited in GenBank under accession numbers MH315874–MH315900. In both the ML and BI analyses, the Moroccan specimens clustered with the three previously described species and the two new species described below. ABGD analysis confirmed these assignments. Based on ca. 400 bp obtained from their paratypes, *M.bakeri* and *M.tingitana* were recognized as potentially different sister species. The additional sampling effort made for this study also extends the distribution range of *M.targouasensis* from southern to central Morocco.

**Figure 2.**
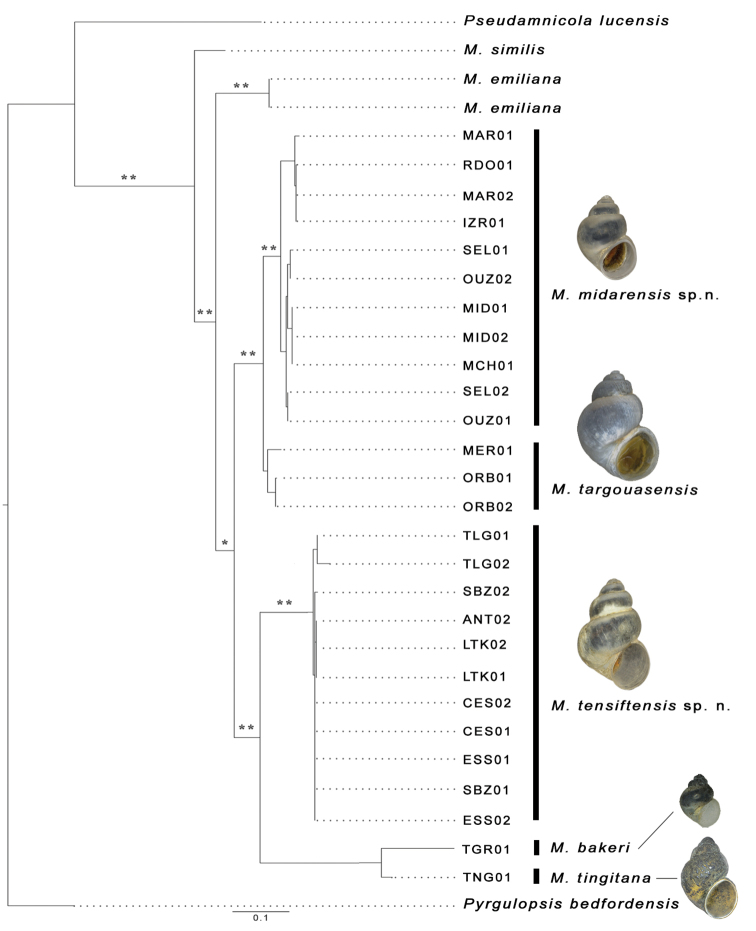
Maximum Likelihood tree based on mtCOI sequences of seven *Mercuria* species. One asterisk represents ML bootstrap values below 75% and BPPs above 0.9; two asterisks represent ML bootstrap values above 75% and BPPs above 0.9. Black bars on the right denote species assignments. Scale bar: expected change per site.

ML and BI topologies were congruent and thus only the ML topology is depicted in Figure 2. This topology showed seven morphologically distinct clades/lineages within *Mercuria*. Moroccan *Mercuria* species clustered together with *M.emiliana* from Spain. This clade was well supported by both ML and BI (75% and 0.95, respectively). All Moroccan specimens formed monophyletic groups, which corresponded to the previously described species and to those described here, except those of *M.targouasensis*. Basal relationships within the *Mercuria* clade were better supported in BI (BPP = 0.93) than in ML (bootstrap value 63%). Within this clade, the species from the Rif Mountains, *Mercuriamidarensis* sp. n., and the Atlas Mountains, *M.targouasensis*, clustered together as sister species (85% in ML and 1.00 in BI), as did the species from the northern Moroccan Atlantic coast: *M.tensiftensis* sp. n., *M.tingitana*, and *M.bakeri* (90% in ML and 1.00 in BI). The latter two species were poorly supported as sisters (67% in ML and 0.81 in BI).

*Mercuriamidarensis* sp. n. and *M.targouasensis* differed from each other by 2.8% (mean sequence divergence, see Table 2). Mean sequence divergence within this first species was 1.8% (0%–3.4%) and 1.3% (1.3%–1.3%) within the second species. Mean sequence divergence between *M.tingitana* and *M.bakeri* was 3.4%. These two species differed from *M.tensiftensis* sp. n., which shows the lowest intraspecific variation (0%), by 4.7%.

**Table 2.** Distance matrix of mean COI sequence divergence (uncorrected p-distances in percentage) within species (on diagonal) and among *Mercuria* species and the selected outgroups (bellow diagonal).

	**1**	**2**	**3**	**4**	**5**	**6**	**7**	**8**	**9**
1. *Pyrgulopsisbedfordensis*	0.0								
2. *Pseudamnicolalucensis*	13.3	0.0							
3. *Mercuriaemiliana*	14.2	15.0	0.0						
4. *M.similis*	14.2	13.3	7.7	0.0					
5. *M.targouasensis*	14.4	14.6	7.4	4.2	1.3				
6. *M.midarensis* sp. n.	13.5	15.2	7.6	6.0	2.8	1.8			
7. *M.tensiftensis* sp. n.	14.6	15.5	7.7	6.4	6.0	6.9	0.0		
8. *M.bakeri*	15.9	16.3	7.3	6.4	4.8	6.8	4.7	0.0	
9. *M.tingitana*	16.7	15.9	8.2	7.3	6.5	8.5	4.7	3.4	0.0

### Systematic descriptions

#### Family Hydrobiidae Stimpson, 1865

##### Genus *Mercuria* Boeters, 1971

###### Mercuria bakeri

Taxon classificationAnimaliaLittorinimorphaHydrobiidae

Glöer, Boeters & Walther, 2015

####### Examined material.

MOROCCO. Paratype (one male): UGSB 18986, Taghramt, 3.5 km towards N, Tangier-Titouan, 23/03/2014 (35°48.912'N, 5°27.618'W).

####### Revised diagnosis.

Shell ovate-conic; periostracum greyish; body whorl large, convex, occupying approximately 3/4 of total shell length; aperture ovate and complete; umbilicus narrow, not covered by the inner lip; central radular tooth formula (3)4–C–4(3)/1–1; bursa copulatrix elongate; bursal duct shorter than bursa length; one seminal receptacle fingerlike, with a short duct; penis strap-like, pigmented from brown to dark grey; penial appendix unpigmented, shorter than penis, base wide, medially positioned on inner edge of penis; nervous system moderately concentrated (mean RPG ratio = 0.40).

####### Description.

Shell ovate-conic (Figure 2), height 3.0–3.5 mm (Glöer et al. 2015). Periostracum greyish. Teleoconch whorls convex, separated by deep sutures. Body whorl occupying approximately 3/4 of total shell length. Umbilicus narrow, partially covered by the inner lip. Aperture ovate, complete, in contact with the body whorl; inner lip thicker than outer lip.

**Figure 3.**
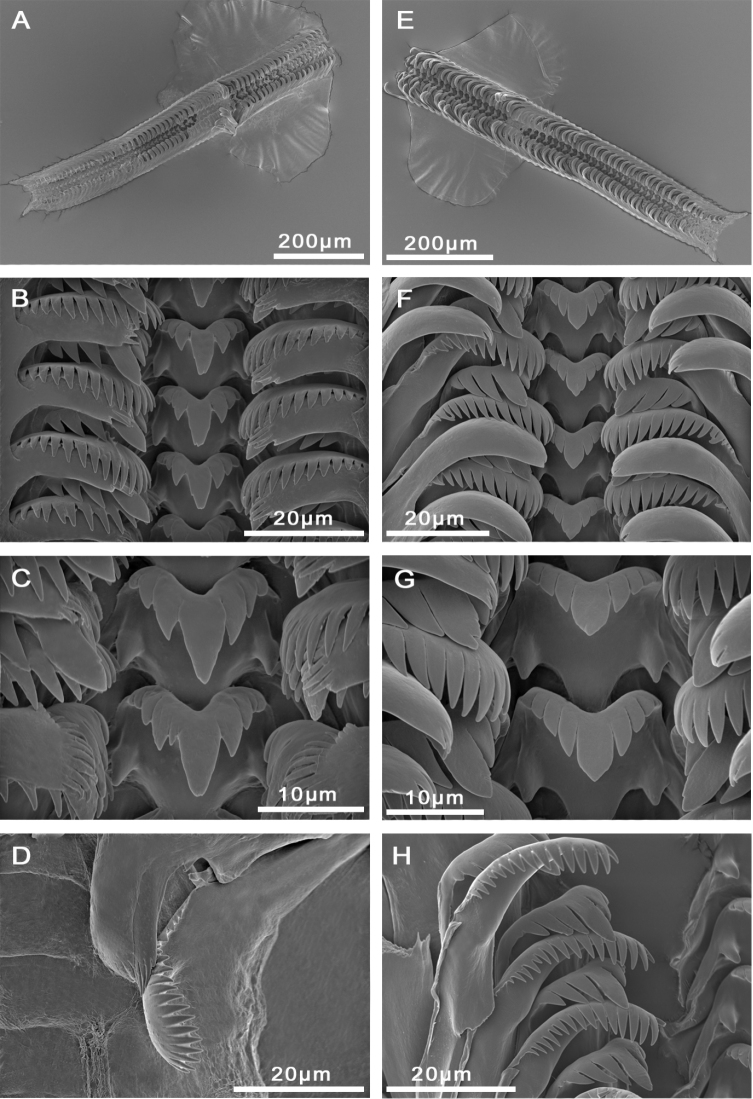
Radulae, **A–D***M.bakeri*, Taghramt **E–H***M.tingitana*, Tangier at 11 km towards Ksar es Seghir. **A, E** Radulae ribbons **B, F** Overview of rows of radulae teeth **C, G** Central tooth **D** Outer marginal tooth **H** Inner marginal and lateral teeth.

Radula with approximately 65 rows of teeth (Figure 3A), medium sized (23% total shell length), 7.5 times longer than wide. Central tooth formula (3)4–C–4(3)/1–1; central cusp long, V-shaped. Lateral tooth formula 3–C–3; central cusp long, V-shaped (Figure 3B, C). Inner and outer marginal teeth having approx. 16 and 20 pointed cusps, respectively (Figure 3D).

**Figure 4.**
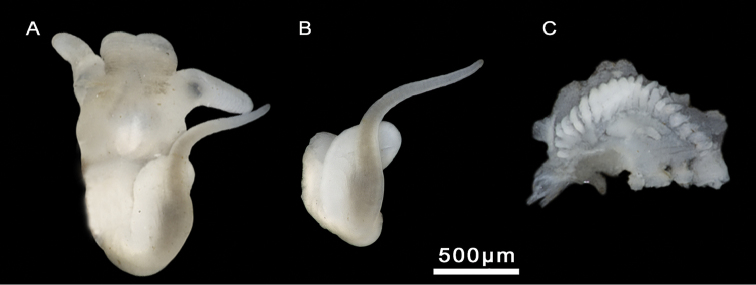
Male genitalia and ctenidium, *M.bakeri*, Taghramt. **A** Head with penis **B** Penis **C** Ctenidium.

Head brown pigmented except for white patches surrounding tentacles and eyes (Figure 4A). Ctenidium well-developed, with ca. 21 gill filaments, occupying most of pallial cavity; osphradium elongate, positioned middle of ctenidium (Figure 4C). Bursa copulatrix elongate. Bursal duct shorter than bursa length. Seminal receptacle fingerlike, with a short duct, joining renal oviduct above the insertion point with bursal duct (based on Glöer et al. 2015). Penis strap-like, attached to central area of head. Penis brown to dark grey pigmented. Penial appendix and base of penis unpigmented. Penial appendix shorter than penis, base wide, medially positioned on inner edge of penis (Figure 4B). Terminal gland large, occupying the whole distal end of the appendix.

####### Distribution.

Only known from the type locality.

####### Remarks.

Based on a short fragment of the mitochondrial COI gene, our phylogenetic analysis depicts *M.bakeri* as sister to *M.tingitana* with a mean uncorrected sequence divergence of 3.4%. A greater genetic distance is not unexpected when including longer sequences. Despite this relatively low genetic distance, morphological differences between these two species are striking, especially in terms of penis shape (penis slender, 3.5 times longer than appendix in *M.bakeri* and penis and appendix almost equal in size in *M.tingitana*) and seminal receptacle (longer in *M.bakeri* than *M.tingitana*).

####### Ecology.

See Glöer et al. (2015).

###### Mercuria tingitana

Taxon classificationAnimaliaLittorinimorphaHydrobiidae

Glöer, Boeters & Walther, 2015

####### Examined material.

MOROCCO. Paratypes (two females): UGSB 17658, Tangier at 11 km towards Ksar es Seghir, 22/03/2014 (35°49.008'N, 5°43.643'W).

####### Revised diagnosis.

Shell ovate-conic, whorls 4–5; periostracum greyish; body whorl large, convex, approx. three-quarters of shell length; aperture ovate and complete; umbilicus narrow, not covered by the inner lip; central radular tooth formula 4–C–4/1–1; bursa copulatrix pyriform to elongate, with a short duct; one seminal receptacle elongate, without duct; penis small, black pigmented; penial appendix as long as penis, slightly pigmented, base wide, distally positioned on inner edge of penis.

####### Description.

**Figure 5.**
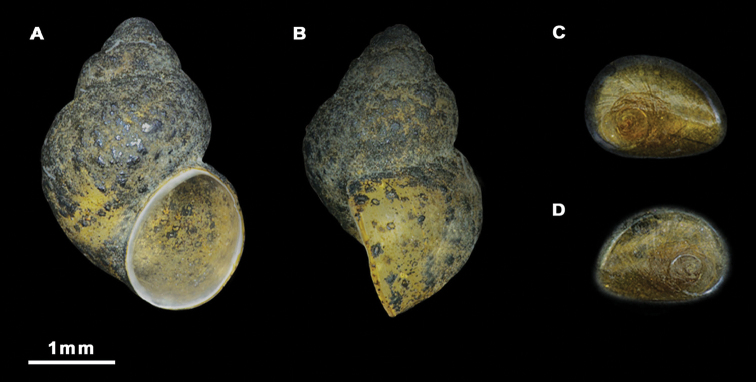
Shells and opercula, *M.tingitana*, Tangier at 11 km towards Ksar es Seghir. **A, B** Shells, **C, D** Opercula (inner, outer sides).

Shell ovate-conic, whorls 4–5, height 2.9–3.6 mm (Figure 5A; Suppl. material 1: Table 1). Body whorl large, approx. two-thirds total shell length. Teleoconch whorls shouldered, separated by deep sutures. Periostracum greyish. Aperture ovate, complete, in contact with the body whorl; inner lip thicker than outer lip; peristome margin straight (Figure 5B). Umbilicus narrow, partially covered by the inner lip.

Operculum as for genus, brownish, whorls 2.5. Muscle attachment area oval located near the nucleus (Figure 5C, D). Radula length intermediate, approx. 600 µm long (20% total shell length), six times longer than wide, with 50–60 rows of teeth (Figure 3E; Suppl. material 1: Table 2). Central tooth formula 4–C–4/1–1; central cusps tongue-shaped. Lateral tooth formula 3–C–3; central cusp long, V-shaped (Figure 3F–H). Inner marginal teeth with 15–16 pointed cusps. Outer marginal teeth with 21–23 cusps (Suppl. material 1: Table 2).

**Figure 6.**
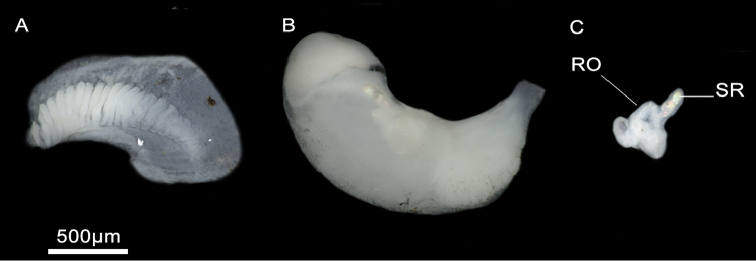
Ctenidium and female genitalia, *M.tingitana*, Tangier at 11 km towards Ksar es Seghir. **A** Ctenidium **B** Pallial oviduct **C** Seminal receptacle (**SR**) and renal oviduct (**RO**).

Animal black pigmented except for neck and eye lobes. Ctenidium well-developed, with 19–23 gill filaments, occupying almost entire length of the pallial cavity. Osphradium elongate, positioned posterior to middle of ctenidium (Figure 6A). Stomach nearly as long as wide; style sac slightly shorter than stomach and surrounded by an unpigmented intestine (Suppl. material 1: Table 3). Glandular oviduct approx. three times longer than wide. Capsule gland shorter and thicker than albumen gland. Renal oviduct coiled, unpigmented. Bursa copulatrix pyriform to elongate, with a short duct. Seminal receptacle small, elongate, sessile, joining renal oviduct above the insertion point with bursal duct (Figure 6B, C; Suppl. material 1: Table 4). Penis attached to central area of head. Penis and penial appendix almost equal in length. Penis black pigmented, short, and triangular. Penial appendix and base of penis unpigmented. Terminal gland small (see Glöer et al. 2015).

####### Distribution.

Only known from the type locality.

####### Remarks.

*Mercuriatingitana* can be distinguished from its congeners in northern Africa by its slender shell and short penis. Genetically, the closest species to *M.tingitana* is *M.bakeri*. However, both these are the most distant species to other Moroccan congeners with an uncorrected pairwise distance range of 4.7–8.5% for *M.tingitana* and of 4.7–6.8% for *M.bakeri*. *Mercuriamidarensis* sp. n. and *M.emiliana* are the most genetically distant from *M.tingitana* according to COI divergences of 8.5% and 8.2%, respectively (Table 2).

####### Ecology.

See Glöer et al. (2015).

###### Mercuria targouasensis

Taxon classificationAnimaliaLittorinimorphaHydrobiidae

Glöer, Boeters & Walther, 2015

Mercuria confusa Backhuys & Boeters, 1974: 113

####### Material.

**Examined material.** MOROCCO. MHNM 18 ZTMH10, UGSB 17912, Oum Rbii Springs, N of Khenifera, 01/06/2015 (33°3.2059'N, 5°24.8797'W); MHNM 18 ZTMH11, UGSB 17955, a small ditch in Mirleft, 02/02/2015 (29°35.0167'N, 10°1.845'W).

####### Revised diagnosis.

Shell ovate-conic, whorls 3–5; periostracum whitish; body whorl occupying more than three-quarters of total shell length; aperture ovate; umbilicus narrow, partially covered by the inner lip; operculum brownish to slightly orange; central radular tooth formula 3–C–3/1–1; bursa copulatrix elongate, with a short duct; one seminal receptacle pyriform, with a short duct; penis gradually tapering, grey; penial appendix shorter than penis, grey, base wide, medially positioned on inner edge of penis; nervous system extremely elongated (mean RPG ratio = 0.70), gently black pigmented.

####### Description.

**Figure 7.**
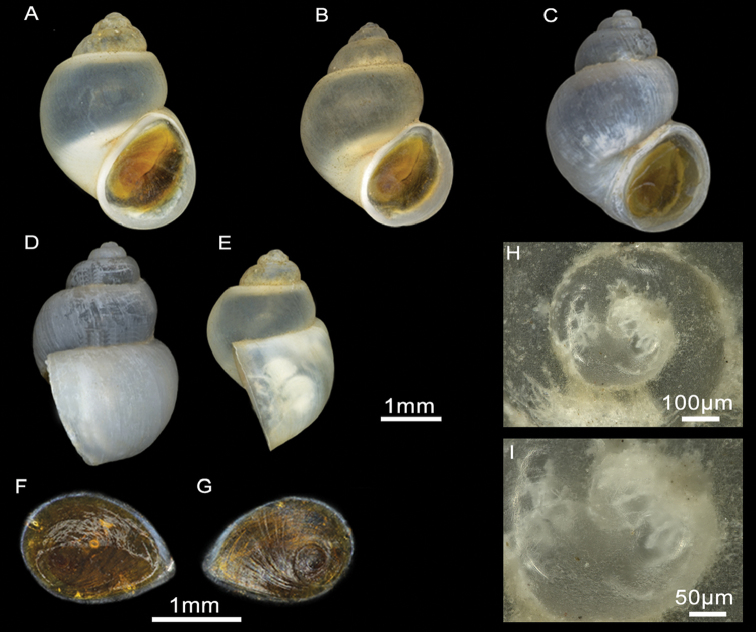
Shells and opercula, *M.targouasensis*. **A, B, E** Shell, Oum Rbii Springs **C, D** Shell, a small ditch in Mirleft **F, G** Opercula (inner, outer sides), Oum Rbii Springs **H, I** Protoconch and detailed microsculpture of protoconch, Oum Rbii Springs.

Shell ovate-conic, whorls 3–5, height 2.63–3.43 mm (Figure 7A–C; Suppl. material 1: Table 1). Periostracum whitish. Protoconch with two whorls, diameter ca. 600 µm; nucleus ca. 140 µm wide (Figure 7H); protoconch microsculpture granulated (Figure 7I). Teleoconch whorls convex, separated by deep sutures. Body whorl large, occupying three-quarters of total shell length. Aperture ovate, complete, in contact with the body whorl; inner lip thicker than outer lip; peristome margin straight (Figure 7D, E). Umbilicus narrow, partially covered by the inner lip.

**Figure 8.**
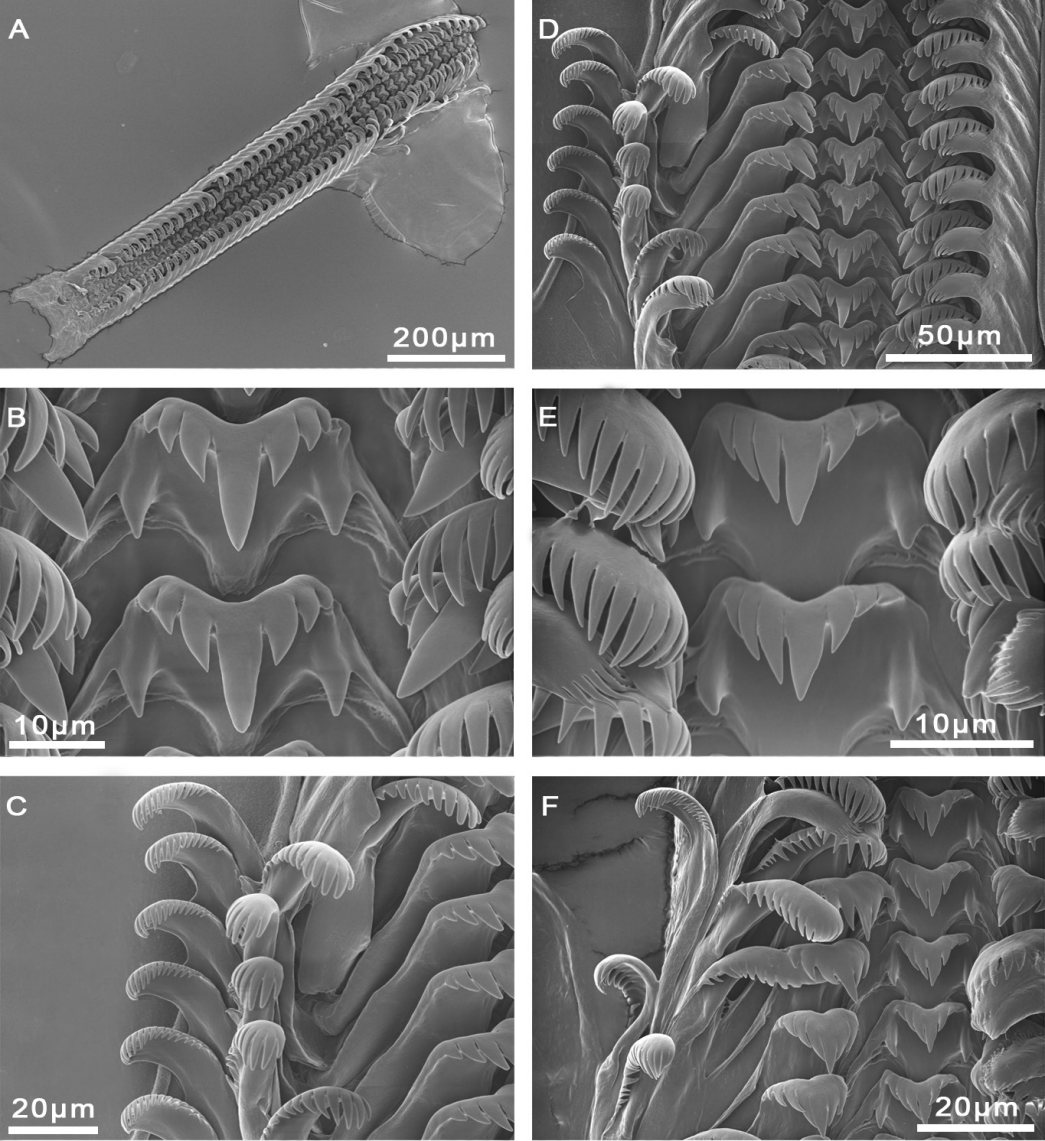
Radulae, *M.targouasensis*. **A–D** Oum Rbii Springs **E, F** A small ditch in Mirleft. **A** Radular ribbon **B, E** Central tooth **C, D, F** Inner marginal, outer marginal, and lateral teeth.

Operculum as for genus, light orange to brown, whorls 2; muscle attachment area near nucleus (Figure 7F, G). Radula length intermediate, ca. 800 µm long (20% total shell length), seven times longer than wide, with approx. 50 rows of teeth (Fig. 8A; Suppl. material 1: Table 2). Central tooth formula 3–C–3/1–1; central cusp tapered, long. Lateral teeth formula (4)3–C–3(4); central cusp wide, V-shaped (Figure 8B, D–F). Inner and outer marginal teeth having 11–15 and 14–18 cusps, respectively (Figure 8C, F).

**Figure 9.**
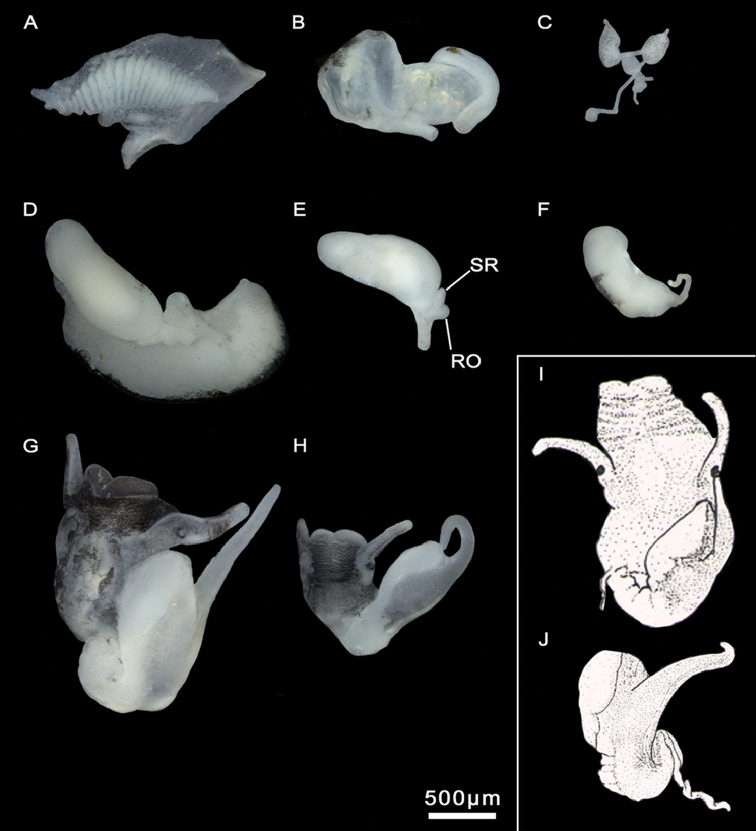
Anatomical structures, *M.targouasensis*. **A–F, H** A small ditch in Mirleft **G, I–J** Oum Rbii Springs. **A** Ctenidium **B** Stomach **C** Partial nervous system **D** Pallial oviduct **E** Bursa copulatrix and seminal receptacle **F** Prostate gland **G, H** Head with penis **I, J** Head and penis drawings. **RO** renal oviduct **SR** seminal receptacle.

Animal black pigmented except for pale area surrounding eye lobes and neck (Figure 9G, H). Ctenidium well-developed, with 19–25 gill filaments, occupying almost entire length of the pallial cavity. Osphradium elongate, positioned approximate middle of ctenidium (Figure 9A). Stomach nearly as long as wide; style sac slightly shorter than stomach, surrounded by an unpigmented intestine (Figure 9B; Suppl. material 1: Table 3). Glandular oviduct three times longer than wide. Capsule gland longer and thicker than albumen gland. Bursa copulatrix pyriform to elongate, with a short duct. Renal oviduct unpigmented, coiled, making three loops. Seminal receptacle pyriform, with a short duct, joining renal oviduct above the insertion point with bursal duct (Figure 9D, E; Suppl. material 1: Table 4). Prostate gland bean-shaped, ca. 2.5 times longer than wide (Figure 9F; Suppl. material 1: Table 5); seminal duct entering the posterior region; pallial vas deferens emerging close to its anterior edge. Penis gradually tapering, attached to the area close to the right eye. Penial appendix slightly pigmented, shorter than penis, base wide, middle positioned on inner edge of penis. Terminal gland large, occupying the whole distal end of the appendix (Figure 9G–J; Suppl. material 1: Table 5). Nervous system gently pigmented, extremely elongated (mean RPG ratio = 0.70; Suppl. material 1: Table 6); cerebral ganglia equal in size and shape (Figure 9C).

####### Distribution.

This species was found in coastal streams in southwestern Morocco and in a spring-fed habitat in the Middle Atlas.

####### Remarks.

The morphological and anatomical descriptions presented here are based on specimens collected at two sites: one in the Mirleft region, 70 km from the type locality, (i.e., ford Oued Assaka), and another, in a more remote place in the Middle Atlas Mountains. The population collected in the surroundings of Mirleft may correspond to the species *Mercuria* ‘*mirlheftensis*’ (nomen nudum) from the same area suggested by García et al. (2010). However, the name *M.* ‘*mirlheftensis*’ is not valid. Specimens collected in the Mirleft area resemble specimens from the type locality of *M.targouasensis* regarding the shape of the penis and prostate, and also of the female genitalia, especially bursa copulatrix shape. Based on the geographic proximity of these two localities and the similarity in shell and anatomical characters of their specimens, we assigned the population from Mirleft to *M.targouasensis*. Specimens from Oum Rbii (Middle Atlas) were also tentatively assigned to this species based on shell and morphological similarities and a short genetic distance (1.3%) between this and the Mirleft population. However, this assignment needs confirmation in future systematic studies on *Mercuria*, which should include these and other Mediterranean species.

*Mercuriatargouasensis* and *M.midarensis* sp. n. are sister species and differ molecularly by 2.1%–3.4% (mean sequence divergence 2.8%). The two species are close in shell dimensions but differ in other shell features such as the relative size of the body whorl (larger in *M.targouasensis*) or the umbilicus (wider in *M.midarensis* sp. n.). They also differ anatomically; *Mercuriamidarensis* sp. n. has typically a strap-like penis, 2.5 times longer than head length, a small penial appendix with narrow insertion into the penis, and an elongate bursa copulatrix, whereas in *M.targouasensis*, the penis is more often gradually tapering, equal or 1.5 times longer than head length, the penial appendix is larger with a wider insertion, and the bursa copulatrix is pyriform to elongate. These two species also differ in the number of cusps on radular teeth (Suppl. material 1: Table 2).

####### Ecology.

In the new localities of *M.targouasensis*, this species was found attached to stones in a saltwater spring in the Middle Atlas (ca. 1,200 m a.s.l. altitude, and 37.9 PSU, practical salinity unit) and in the sediment of a ditch in the region of Mirleft cohabiting with *Melanopsispraemorsa*.

###### Mercuria midarensis sp. n.

Taxon classificationAnimaliaLittorinimorphaHydrobiidae

http://zoobank.org/C15971DC-9513-4AC8-B7E5-D74D0EFF8644

####### Type material.

Holotype, MNCN 15.05/200019H (Ethanol 80%), a small ditch 7 km from Midar, Northern Morocco, 34°54.5795'N, 3°34.0292'W, 03/06/2015, leg. K.B., M.G., D.D., T.H. Paratypes MNCN 15.05/200019P, UGSB 17921, and MHNM 18 ZTMH12 (from the same lot).

####### Other material.

MOROCCO. MHNM 18 ZTMH20, UGSB 17921, UGSB 17922, a small ditch 7 km from Midar, 03/06/2015 (34°54.5795'N, 3°34.0292'W); MHNM 18 ZTMH13, UGSB 19933, Selouan River, S of Nador, 30/04/2016 (35°4.6117'N, 2°55.485'W); MHNM 18 ZTMH14, UGSB 19939, Ouzej River, Al Aaroui, 30/04/2016, (35°0.3634'N, 2°59.5133'W); UGSB 19935, Cherarba ponds W of Saidia, 28/04/2016 (35°6.3116'N, 2°20.75'W); MHNM 18 ZTMH15, UGSB 19934, Izerouan River, 20 km W of Nador, 12/05/2015 (35°9.8333'N, 3°6.6'W); MHNM 18 ZTMH16, UGSB 19940, Mariouari River, 30/04/2016 (35°18.36'N, 2°58.6483'W); MHNM 18 ZTMH17, UGSB 19938, Messoussate River, Selouan, 02/05/2016 (35°3.81'N, 2°54.383'W). SPAIN. MHNM 18 ZTMH18, UGSB 19937, Rio de Oro, Melilla, 18/05/2015, (35°17.2483'N, 2°56.6283'W).

####### Diagnosis.

Shell ovate-conic, whorls 4–5; periostracum whitish; body whorl large, convex, occupying more than three-quarters of total shell length; aperture ovate, complete; umbilicus narrow, not covered by the inner lip; protoconch microsculpture granulated; operculum dark orange to dark brown; central radular tooth formula (3)4–C–4(3)/1–1; bursa copulatrix elongate, with a short duct; one seminal receptacle elongate without duct; penis gradually tapering to strap-like, light to dark grey pigmented; penial appendix similarly pigmented, shorter than penis, base wide, medially positioned on inner edge of penis; nervous system extremely elongated (mean RPG ratio = 0.70), slightly pigmented.

####### Description.

**Figure 10.**
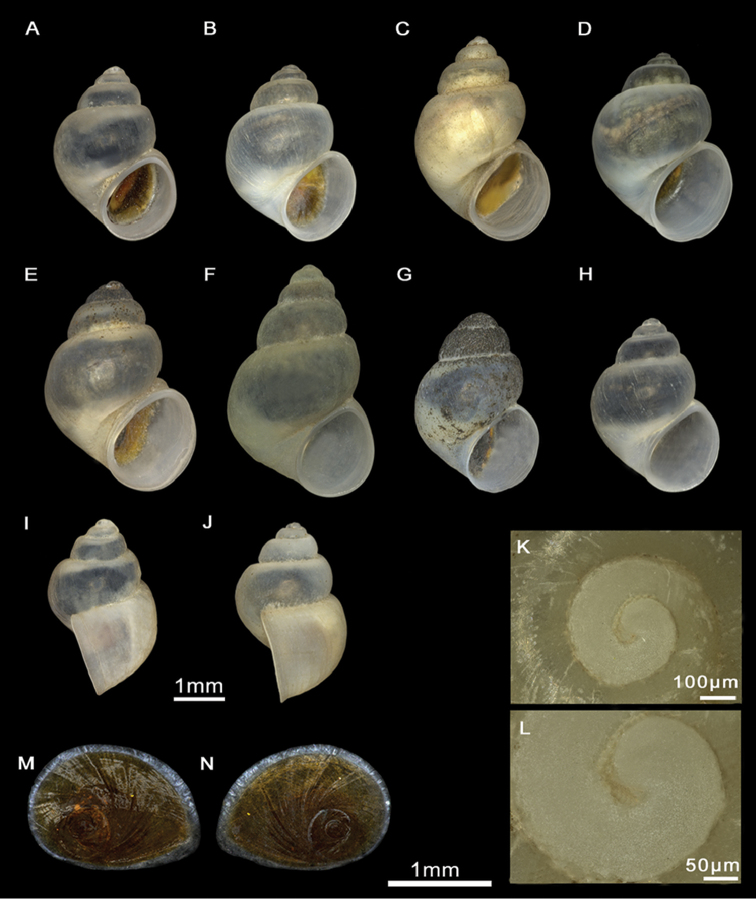
Shells and opercula, *M.midarensis* sp. n. **A, I** Holotype MNCN 15.05/200019H **B** Shell, a small ditch 7 km from Midar **C** Shell, Selouan River **D** Shell, Ouzej River **E** Shell, Cherarba ponds **F** Shell, Mariouari River **G** Shell, Rio de Oro **H** Shell, Izerouan River **M, N** Opercula (inner, outer sides), a small ditch 7 km from Midar **K, L** Protoconch and detailed microsculpture of protoconch, Selouan River.

Shell ovate-conic, whorls 4–5, height 3–4.7 mm (Figure 10A–J; Suppl. material 1: Table 1). Periostracum whitish. Protoconch ca. 400 µm wide, whorls 1.5; nucleus ca. 125 µm wide; protoconch microsculpture granulated (Figure 10K–L). Teleoconch whorls shouldered, separated by deep sutures. Body whorl large, occupying three-quarters of total shell length. Aperture ovate, complete, sometimes in contact with the body whorl; inner lip thicker than outer lip; peristome margin straight (Figure 10I–J). Umbilicus narrow, not covered by the inner lip.

**Figure 11.**
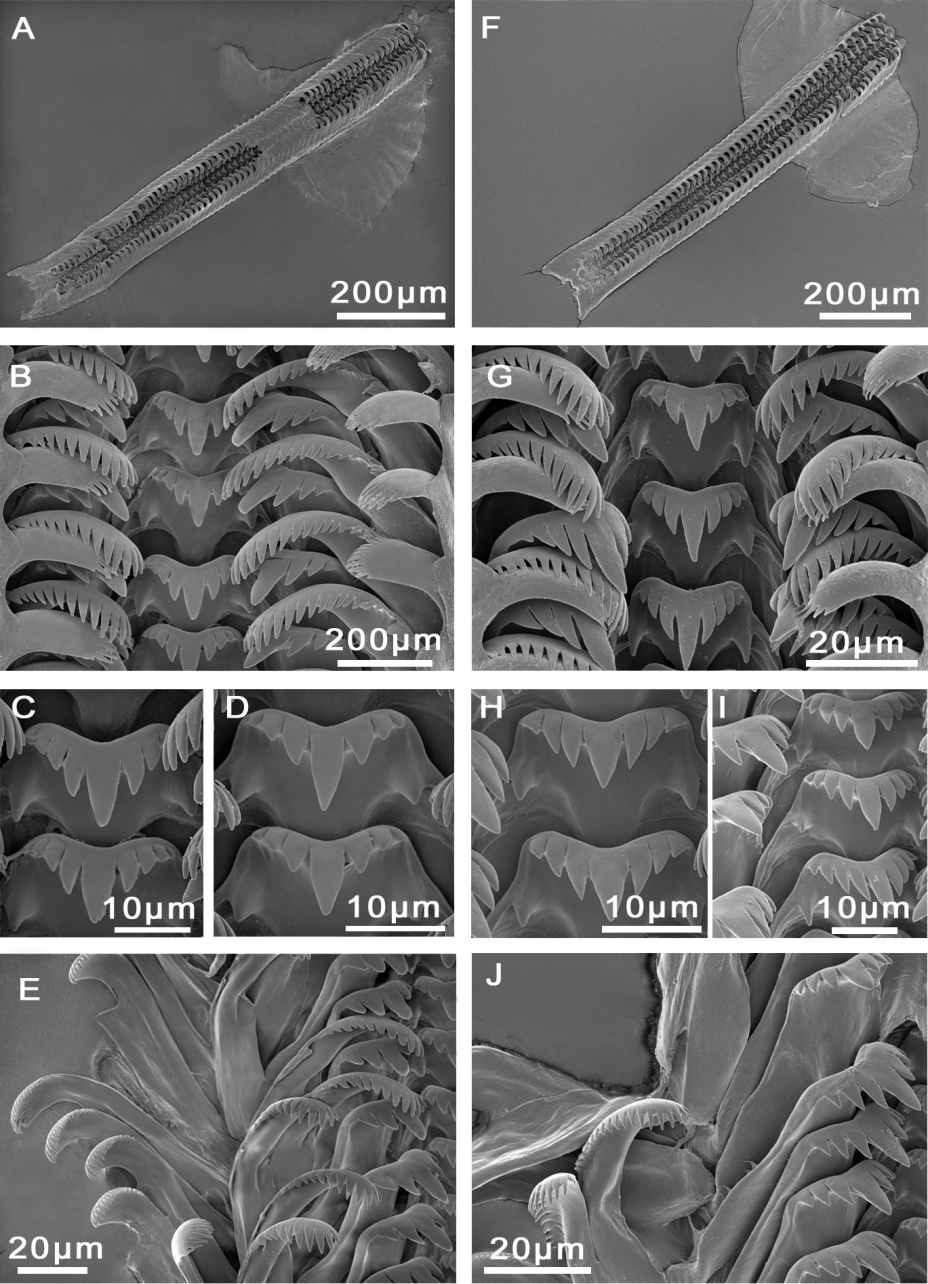
Radulae, *M.midarensis* sp. n. **A–D** Selouan River **E** A small ditch 7 km from Midar **F, H–J** Rio de Oro **G** Izerouan River. **A, F** Radulae ribbons **B, G** Overview of rows of radulae teeth **C, D, H, I** Central tooth **E, J** Inner marginal, outer marginal, and lateral teeth.

Operculum as for genus, dark orange to dark brown, surrounded by a thin and transparent border, whorls 2, muscle attachment area oval and located near the nucleus (Figure 10M–N). Radula length intermediate, ca. 900 µm long (23% total shell length), 7.5 times longer than wide (Figure 11A, F; Suppl. material 1: Table 2); 53–67 rows of teeth. Central tooth formula (3)4–C–4(3)/1–1; central cusp V-shaped, long (Figure 11B–D, G–I). Lateral tooth formula (2)3–C–3(2); central cusp tongue-shaped (Figure 11J). Inner marginal teeth with 12–14 long, pointed, cusps. Outer marginal teeth bearing 18–20 cusps (Figure 11E–J, Suppl. material 1: Table 2).

**Figure 12.**
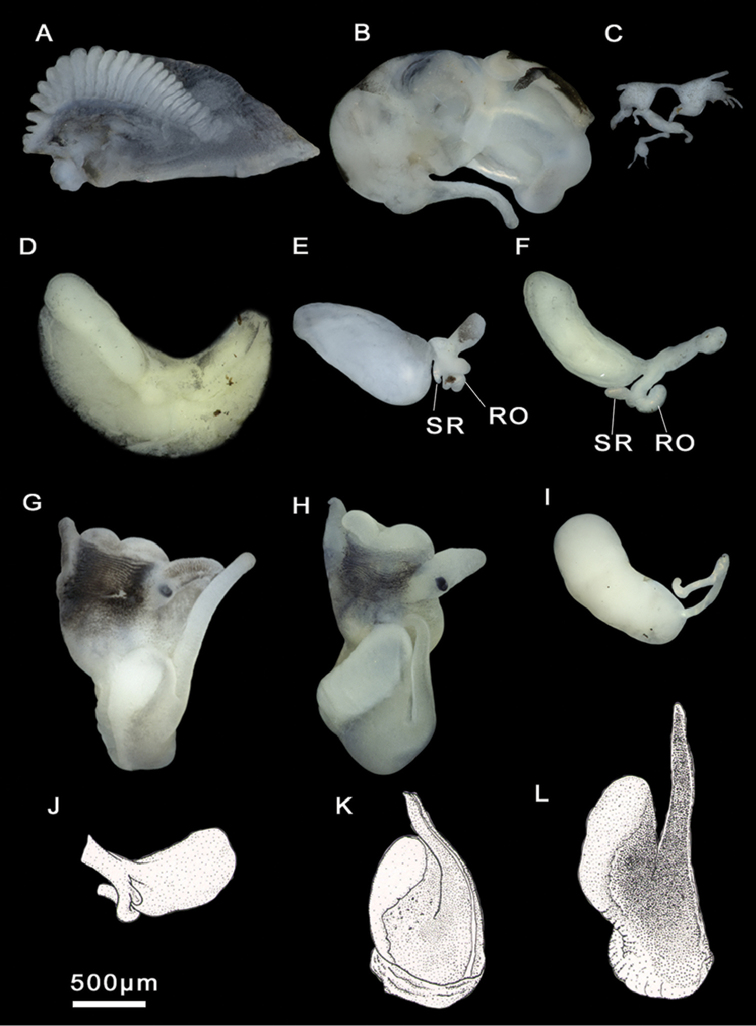
Anatomical structures, *M.midarensis* sp. n. **A–C, E, G, I, K–L** a small ditch 7 km from Midar **D** Ouzej River **F** Rio de Oro **H** Selouan River. **A** Ctenidium **B** Stomach **C** Partial nervous system **D** Pallial oviduct **E, F** Bursa copulatrix and seminal receptacle **J** Bursa copulatrix drawing **G, H** Head with penis **I** Prostate gland **K, L** Penis drawings. **RO** renal oviduct **SR** seminal receptacle.

Head and tentacles dark brown pigmented; eye lobes and snout margin unpigmented; pigmentation lighter on neck (Figure 12G–H). Ctenidium well developed, with 21–26 gill filaments, occupying most of pallial cavity. Osphradium elongate, positioned opposite middle of the ctenidium (Figure 12A). Stomach slightly longer than wide; style sac shorter than stomach, surrounded by an unpigmented intestine (Figure 12B, Suppl. material 1: Table 3). Glandular oviduct from two to three times longer than wide. Albumen gland shorter than capsule gland. Bursa copulatrix elongate, from two to three times longer than wide. Bursal duct very short. Renal oviduct unpigmented, coiled. Seminal receptacle elongate, without duct (Figure 12D–F, J, Suppl. material 1: Table 4). Prostate gland bean-shaped, approx. two times longer than wide (Figure 12I). Seminal duct entering the posterior region and pallial vas deferens emerging close to its anterior edge. Penis gradually tapering to strap-like, attached well behind right eye, base large and slightly pigmented. Penis tapering, light grey pigmented. Penial appendix light grey, shorter than penis, two times longer than wide, base wide, medially positioned on inner edge of penis (Figure 12G–H, K–L; Suppl. material 1: Table 5). Terminal gland large, occupying the whole distal end of the appendix. Nervous system slightly pigmented, extremely elongated (mean RPG ratio 0.70; Suppl. material 1: Table 6); cerebral ganglia almost equal in size and shape (Figure 12C).

####### Etymology.

This species is named *midarensis* after Midar, the nearby city where this species was collected for first time.

####### Distribution.

*Mercuriamidarensis* sp. n. is distributed mostly in spring-fed and riverine habitats of northeastern Morocco and the surroundings of the Spanish city of Melilla.

####### Remarks.

The mean uncorrected sequence divergence within *Mercuriamidarensis* was 1.8%, ranging from 0%–3.4%. Despite their geographic proximity, *M.midarensis* populations were genetically resolved into two geographically separate groups: the northern populations, i.e. those from the Mariouari River, Rio de Oro, and Izerouan River basins; and the southern ones, i.e. ditch in Midar (type locality), Selouan River, Ouzej River, and Cherarba Ponds. Although mean genetic distances between these two groups of populations were relatively high ranging from 2.6% to 3.4% for COI, we found no consistent morphological differences to consider them distinct. However, we did observe shell variability within each group. For most localities, shell length mostly ranged from 3.0 mm to 4.5 mm, with the body whorl occupying 75–82% total shell length. As exceptions, specimens from the Mariouari and Selouan river basins had larger shells (4.0–5.5 mm and 3.6–4.8 mm, respectively) and a body whorl occupying 60–75% total shell length (Figure 10C, F). Although female genitalia morphology and morphometry were similar in all populations of the species, we detected variability in penis shape and size. Both the southern populations and the population from the Rio de Oro basin (within the northern group) are characterized by a long penis (2.1–3.2 mm), 2 to 3 times longer than the appendix and 1.5 to 3 times longer than the head. In contrast, northern populations have a shorter penis (0.6–2.1 mm), which is 2.6 to 4 times longer than the appendix. Moreover, in the population from the Izerouan River basin, the penis is 1.4 to 1.8 times longer than the head while it is shorter than the head in the population from the Mariouari River basin.

*Mercuriabakeri* and *M.tingitana* have more elongate, tall-spired shells than most of the populations of *M.midarensis* sp. n., except for specimens from the Mariouari and Selouan rivers. *Mercuriamidarensis* sp. n. differs from *M.bakeri* in its shorter seminal receptacle, shorter bursal duct, and larger penial appendix, and from *M.tingitana* in its longer seminal receptacle, larger penis, and fewer cusps on the radular teeth. The mean genetic distance between *M.bakeri* and *M.midarensis* sp. n. was 6.8% while between the latter and *M.tingitana* it was 8.5%.

####### Ecology.

Most specimens were found in small ditches or river tributaries attached to stones or simply in the sediment. *Mercuriamidarensis* sp. n. co-occurs with other gastropod species such as *Melanopsispraemorsa*, *Galbatruncatula*, *Ancylusfluviatilis*, and *Physellaacuta*.

###### Mercuria tensiftensis sp. n.

Taxon classificationAnimaliaLittorinimorphaHydrobiidae

http://zoobank.org/42E0CFAF-0F49-48AB-BFC8-9323FEB6996D

####### Type material.

Holotype, MNCN 15.05/200018H (ethanol 80%), a ditch in Sidi Bouzid, Chichaoua, Morocco, 31°29.6133'N, 8°47.1116'W, 28/11/2015, K.B., M.G. Paratypes MNCN 15.05/200018P, UGSB 17910, and MHNM 18 ZTMH4 (from the same lot).

####### Other material.

MOROCCO. MHNM 18 ZTMH19, UGSB 17910, ditch in Sidi Bouzid, Chichaoua, 28/11/2015 (31°29.6133'N, 8°47.1116'W); MHNM 18 ZTMH5, UGSB 17914, a pond near Lahjar Spring, Essaouira, 28/11/2015 (31°38.7583'N, 9°35.0983'W); MHNM 18 ZTMH6, UGSB 17918, ditch in Haddada Bouzerktoun, Essaouira, 28/11/2015 (31°37.95'N, 9°35.0983'W); MHNM 18 ZTMH7, UGSB 19944, ditch in Agadir N’tachraft, 34 km S of Marrakesh, 20/02/2017 (31°23.0917'N, 8°7.353'W); MHNM 18 ZTMH8, UGSB 19945, a spring near Lalla Takerkoust dam, 34 km S of Marrakesh, 20/02/2017 (31°22.5491'N, 8°7.638'W); MHNM 18 ZTMH9, UGSB 19946, Talkount, 80 km E of Marrakesh, 21/02/2017 (31°40.5775'N, 7°16.0298'W).

####### Diagnosis.

Shell ovate-conic, whorls 4–5; periostracum whitish, exceptionally yellowish; body whorl large, convex, occupying approx. two-thirds of total shell length; umbilicus narrow, not covered by the inner lip; aperture ovate; protoconch microsculpture grooved; central radula tooth formula (5)4–C–4(5)/1–1; bursa copulatrix elongate, with a short duct; one seminal receptacle elongate, with a short duct; penis gradually tapering; penial appendix dark pigmented, rectangular, shorter than penis, base narrow and black pigmented, medially positioned on inner edge of penis; nervous system elongated (mean RPG ratio = 0.64), slightly pigmented.

####### Description.

**Figure 13.**
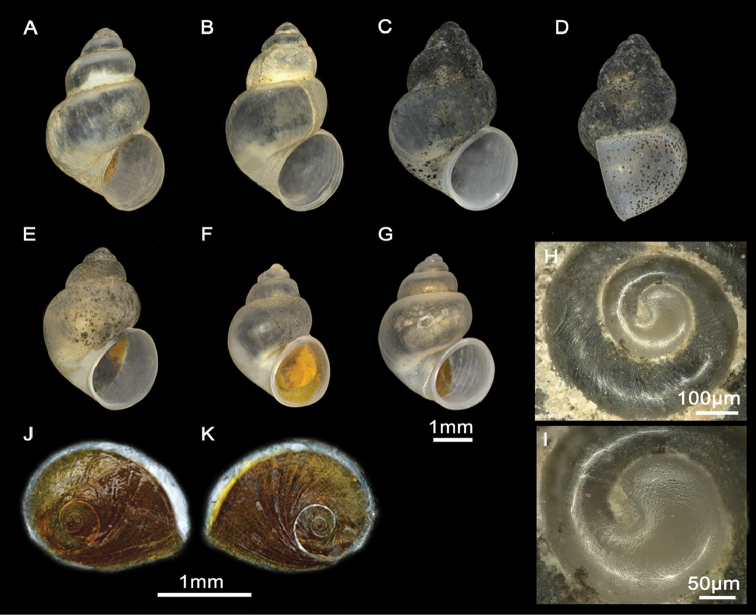
Shells and opercula, *M.tensiftensis* sp. n. **A** Holotype MNCN 15.05/200018H, **B** Shell, ditch in Sidi Bouzid **C, D** Shell, ditch in Agadir N’tachraft **E** Shell, ditch in Talkount **F** Shell, a pond near Lahjar Spring **G** Shell, a spring near Lalla Takerkoust dam **J, K** Opercula (inner, outer sides), ditch in Agadir N’tachraft **H, I** Protoconch and detailed microsculpture of protoconch, ditch in Agadir N’tachraft.

Shell ovate-conic, whorls 4–5, height 3–5.1 mm (Figure 13A–G; Suppl. material 1: Table 1). Periostracum whitish. Protoconch ca. 400 µm wide, whorls 1.5; nucleus ca. 125 µm wide (Figure 13H); protoconch microsculpture grooved (Figure 13I). Teleoconch whorls convex, with deep sutures. Body whorl large, occupying approx. two-thirds of total shell length. Aperture ovate, often attached to body whorl on the top; inner lip thicker than outer lip; peristome margin straight narrow (Figure 13D). Umbilicus narrow, not covered by the inner lip.

**Figure 14.**
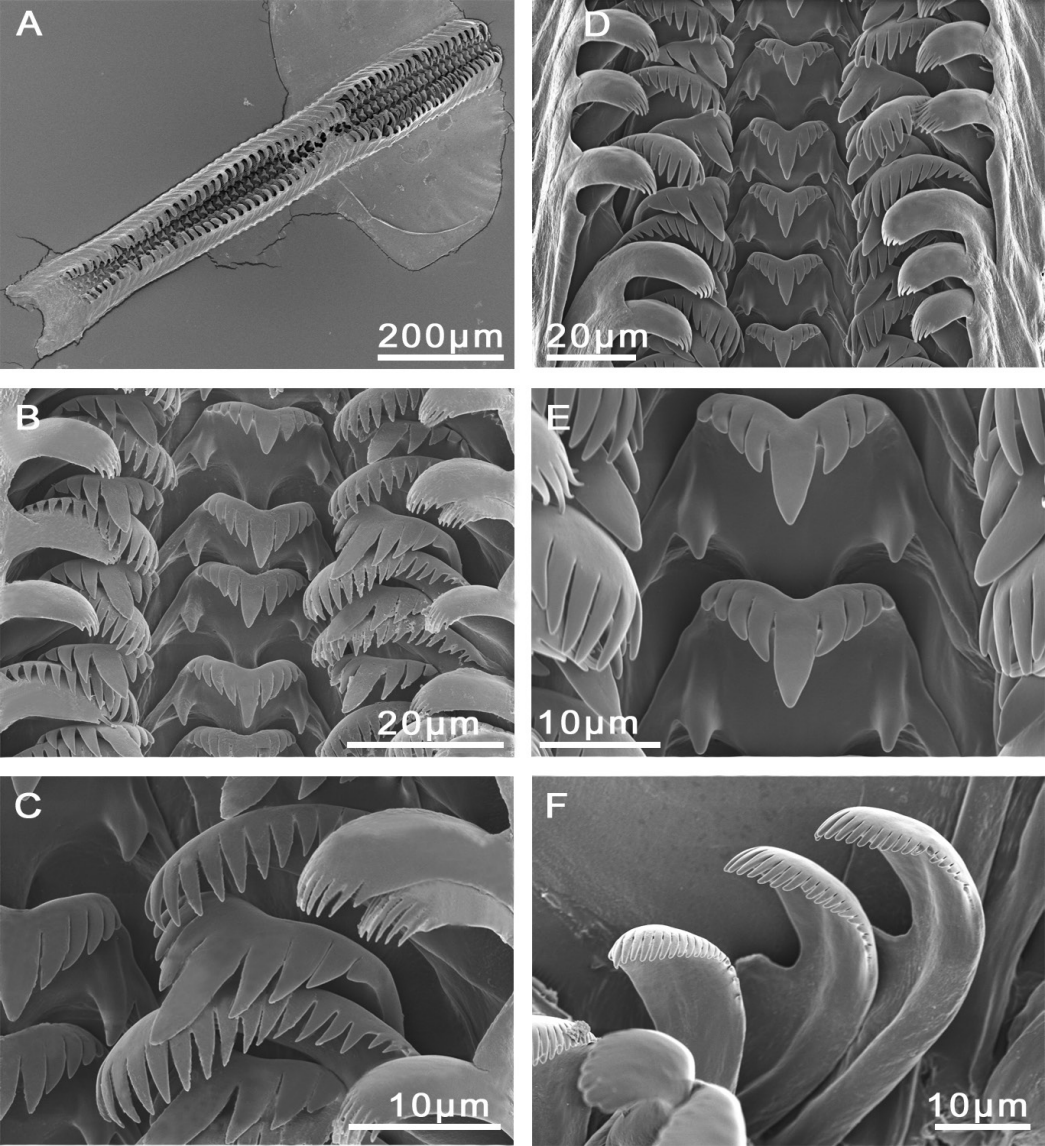
Radulae, *M.tensiftensis* sp. n. **A, E** a spring near Lalla Takerkoust dam **B, C** a pond near Lahjar Spring **D, F** ditch in Sidi Bouzid. **A** Radular ribbon **B, D** Rows of radular teeth **C** Lateral tooth and inner marginal teeth **E** Central tooth **F** Outer marginal teeth.

Operculum as for genus, orange to brownish, about two whorls; muscle attachment area oval and located near the nucleus (Figure 13J–K). Radula length intermediate, ca. 900 µm long (25% total shell length), approx. eight times longer than wide; approx. 60 rows of teeth (Figure 14A). Central tooth formula (5)4–C–4(5)/1–1; central cusp V-shaped (Figure 14B, D, E). Lateral tooth formula (4)3–C–3(4); central cusp long, tongue-shaped (Figure 14B–C). Inner marginal teeth bearing 13–16 cusps and outer marginal with 15–21 cusps (Figure 14D, F; Suppl. material 1: Table 2).

**Figure 15.**
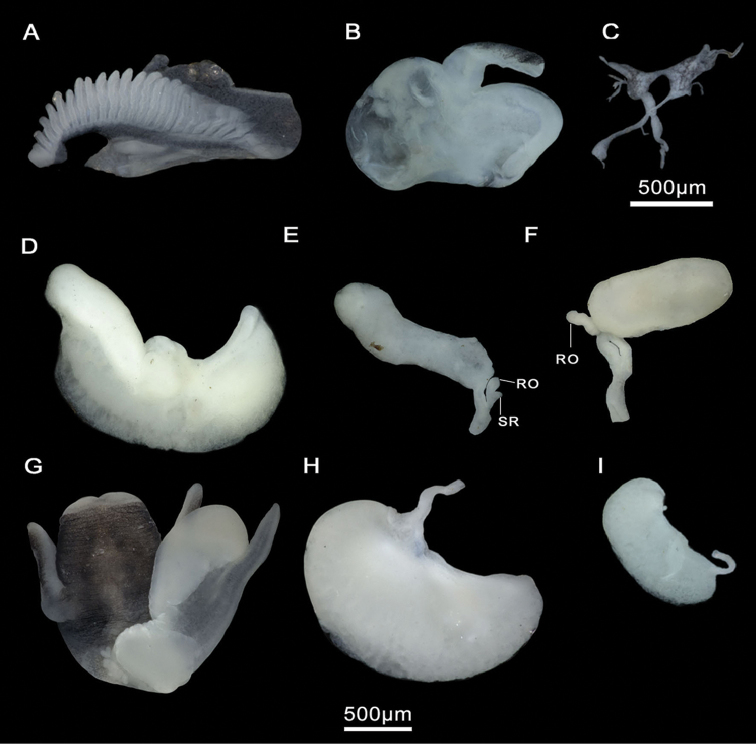
Anatomical structures, *M.tensiftensis* sp. n. **A, D, E, G, H** ditch in Sidi Bouzid **B, F, I** a pond near Lahjar Spring **C** a spring near Lalla Takerkoust dam. **A** Ctenidium **B** Stomach **C** Partial nervous system **D** Pallial oviduct **E, F** Bursa copulatrix and seminal receptacle **G** Head with penis **H, I** Prostate gland. **RO** renal oviduct **SR** seminal receptacle.

Animal darkly pigmented except for neck and tentacles (Figure 15G). Ctenidium well-developed, with 23–27 gill filaments, occupying nearly entire length of pallial cavity. Osphradium elongate, positioned opposite middle of ctenidium (Figure 15A). Stomach slightly longer than wide, with two chambers almost equal in size; style sac longer than wide, surrounded by unpigmented intestine (Figure 15B; Suppl. material 1: Table 3). Glandular oviduct approx. three times as long as wide. Albumen gland longer than capsule gland (Figure 15D–F). Bursa copulatrix elongate, two to three times longer than wide, with a duct shorter than bursal length. Renal oviduct unpigmented, coiled, making 2–3 loops. Seminal receptacle elongate, with a short duct, joining renal oviduct just above the insertion point with bursal duct (Figure 15E–F; Suppl. material 1: Table 4). Prostate gland approx. two times longer than wide, bean-shaped; seminal duct entering the posterior region; pallial vas deferens emerging close to its anterior edge. Penis gradually tapering, attached to the area close to the right eye. Penis dark pigmented, tapering. Penial appendix dark pigmented, shorter than penis, base narrow, medially position on inner edge of penis. Terminal gland occupying the whole distal end of the appendix (Figure 15G–I; Suppl. material 1: Table 5). Nervous system with black granules, elongate (mean RPG ratio 0.64; Suppl. material 1: Table 6); cerebral ganglia approx. equal in size; ganglia darker than connectives and commissures (Figure 15C).

####### Etymology.

The name *tensiftensis* refers to the hydrological basin (Tensift) where this species was collected.

####### Distribution.

This species was found in ponds, springs, and ditches in proximal localities of the Tensift River basin in northwestern Morocco.

####### Remarks.

Shells of this species vary in size (2.4–5.1 mm shell height) and, accordingly, two morphotypes can be distinguished in all populations. One morphotype comprises small to medium-sized shells (2.4–4.0 mm shell height), with slightly shouldered spire whorls and a thick aperture. This morphotype is found in Lahjar, Talkount, and Lalla Takerkoust with an average shell length of 3.5 mm. The second larger group (4.0–5.1 mm shell height) comprises shells with five sloping spire whorls and a thin aperture. This morphotype is well represented in the populations from Sidi Bouzid Springs and Agadir N’tachraft with an average shell length of 4.1 mm. Despite this morphological variation within the species, the estimated genetic distance was 0% for COI.

**Figure 16.**
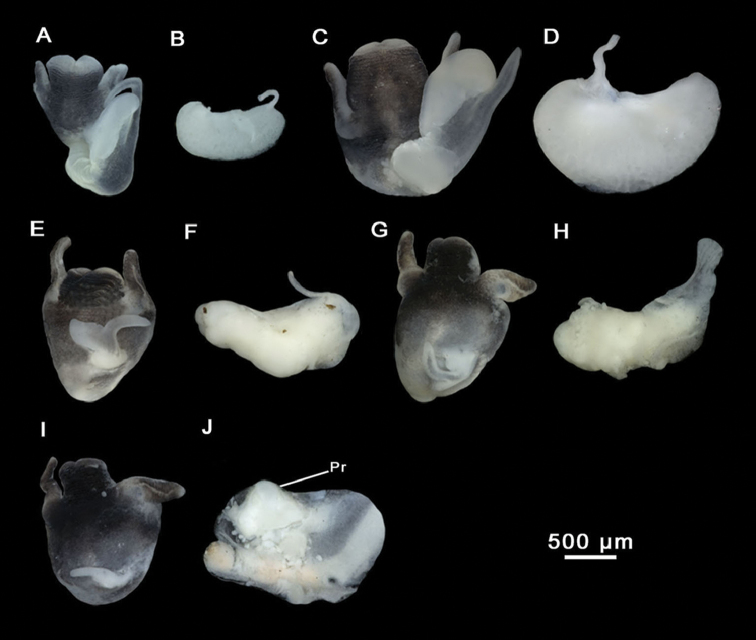
Head and prostate glands of non-parasitized and parasitized males, *M.tensiftensis* sp. n. **A, B** Non-parasitized male, a spring near Lalla Takerkoust dam **C, D** Non-parasitized male, ditch in Sidi Bouzid **E, F** Parasitized male, ditch in Sidi Bouzid **G–J** Parasitized males, ditch in Agadir N’tachraft. **Pr** prostate gland.

Two morphotypes of male reproductive organs were also observed in dissected specimens. The most represented is that with a long penis, large appendix, and large prostate gland (localities of Lahjar near Essaouira, Sidi Bouzid, and Lalla Takerkoust dam). However, other dissected males showed a smaller retracted penis and a small degraded prostate gland (localities of Agadir N’tachraft and Talkount). We observed that this second group of males contained parasites known to cause castration in host snails (Lim and Heyneman 1972, Combes and Cheng 1986, Mouahid and Mone 1990, Ashby and Gupta 2014) and propose this as the cause of such variation (see Figure 16). According to our observations in parasitized specimens of different populations, male and female genitalia seem more affected than other organs.

*Mercuriatensiftensis* sp. n. is characterized by its long shell (the longest shells among Moroccan *Mercuria* species) and its large and gradually tapering penis with a terminal gland occupying the entire distal end of the penial appendix. The new species differs from *M.midarensis* sp. n. in its shorter penis (two times vs. three times longer than appendix in *M.tensiftensis* sp. n. and *M.midarensis* sp. n., respectively) (Suppl. material 1: Table 5), from *M.targouasensis* in its more elongate bursa copulatrix (Suppl. material 1: Table 4) and from *M.similis* in its longer shell and its larger and longer penis. These morphological and anatomical differences were supported by molecular data. Hence, the mean genetic distance between *M.tensiftensis* sp. n. and *M.similis* was 6.4% and between the former and *M.targouasensis* and *M.midarensis* sp. n. were 6.0% and 6.9%, respectively.

####### Ecology.

*Mercuriatensiftensis* sp. n. was found in ditches used for irrigation, springs, and ponds, attached to stones or dead branches in the water. Most of these localities, including the type one, are small water bodies under risk of desiccation or destruction. Co-occurring species were *Galbatruncatula*, *Melanopsispraemorsa*, and *Physellaacuta*.

## Discussion

This study provides the first molecular phylogenetic data on congeners of the genus *Mercuria* along with taxonomic descriptions of previously unknown anatomical structures for this genus such as the radula or nervous system. By integrating both molecular and morphological data, we extended the morphological information available for the three previously identified *Mercuria* species from Morocco (Glöer et al. 2015), described two new species from this region, and revealed phylogenetic relationships between these species and the European *M.emiliana* and *M.similis*. All species were recovered as independent entities in our phylogenetic inference (Figure 2) and ABGD analyses, including the European species with an uncorrected p-distance of 7.7% between them. These findings contradict previous studies (Girardi 2004, Gargominy et al. 2011, Boeters and Falkner 2017) in which the populations of *M.emiliana* studied by Boeters (1988) were synonymized with *M.similis* and the populations of *M.emiliana*described by Paladilhe (1866) were included within the genus *Pseudamnicola* Paulucci, 1878. Our molecular study revealed that sequences of *M.emiliana* available in GenBank (Table 1) were genetically distant from both the species *M.similis* and those of the genus *Pseudamnicola* (Table 2). Furthermore, according to these genetic results, neither the species *M.similis* nor *M.emiliana* were detected among our hydrobiid populations collected in Morocco. However, only genetic surveys and anatomical examinations of specimens from the type localities of both species will help resolve this taxonomic controversy. Since our genetic analyses included specimens collected from different sites than the type localities, we are unable to confirm or reject this synonymy.

In contrast with the low sequence divergence found among populations of *Mercuriatensiftensis* sp. n. (0% divergence), a higher mtDNA variation within *M.midarensis* sp. n. and *M.targouasensis* (0%–3.4% and 0%–1.3% divergence, respectively) was observed. Although an understanding of the biogeographical splitting processes of these species is beyond the scope of this study, we associate these wide ranges of sequence divergence within the latter two species with the high tectonic activity of the Rif and Atlas regions. Our data also point to remarkable morphological and anatomical variation within *Mercuria* species (e.g., within *M.tensiftensis* sp. n.), especially in shell shape and size, and penis and radula features, which may have been caused not only by adaptation (genetic or plastic) but also by seasonality and parasitism.

Shell growth differentially influenced by environmental conditions could result in different morphotypes within a species (Urdy et al. 2010). This has been recently shown for the freshwater gastropod species *Potamopyrgusantipodarum* (Gray, 1843) (Kistner and Dybdahl 2014; Verhaegen et al. 2018). Thus, given that *M.tensiftensis* sp. n. and *M.midarensis* sp. n. were found in a wide spectrum of habitat types, variation in shell shape and size within these species could be a response to variable conditions. A potential adaptive value of morphological variation within *Mercuria* species should be better evaluated in further common garden studies (see, for instance, Verhaegen et al. 2018). Lengths of the penis and the penial appendix were shown, however, to vary within *Mercuria* species according to the sexual maturity of the individuals, regardless of shell size. Holyoak et al. (2017) observed that most males across different populations of the species *M.tachoensis* (Frauenfeld, 1865) presented larger penial appendices from November to May, likely coinciding with their annual reproductive period. Another factor that can mislead taxonomists about intrinsic anatomical variation is the presence of parasites. We found that parasitized males of *M.tensiftensis* sp. n. had a smaller penis and prostate gland than non-parasitized ones. Indeed, several studies have shown that some parasites may lead to castration of the host mollusc (Lim and Heyneman 1972, Combes and Cheng 1986, Mouahid and Mone 1990, Ashby and Gupta 2014).

All these sources of variability suggest that the most efficient approach to delimit and identify *Mercuria* species is the integrated analysis of morphological descriptions and genetic data. Accordingly, when delimiting the species of this genus, intraspecific morphological differentiation of *Mercuria* species should be treated with caution and additionally assessed through molecular evidence.

Uncorrected pairwise distances between the *Mercuria* species examined here ranged from 2.8% to 8.5% with an average of 6.3%, which is lower than averages described for other spring hydrobiids, such as *Corrosella* Boeters, 1970 (5.3–12% average 9% in Delicado et al. 2013) or *Pyrgulopsis* Call & Pilsbry, 1883 (2.8–11.2% in Hershler et al. 2003), though higher than among species of the brackish-water genus *Hydrobia* Hartman, 1821 (average of 4.5%, Wilke et al. 2000). Note that genetic distances between *Mercuria* species are more comparable to those calculated for the genus *Pseudamnicola* (0.5–10% average 6.7% in Delicado et al. 2015), which could be attributed to their similar habitat preferences (i.e., small lowland rivers and streams). This common ecological pattern is suggested by numerous records of these co-occurring genera in Algeria (Glöer et al. 2010), Malta (Glöer et al. 2015), Spain (Boeters 1988), and Morocco (present study). However, although most of the *Mercuria* populations from Morocco were found in low-lying areas, some populations inhabited the Atlas Mountains, indicating a wide habitat range for this genus. Additional field surveys and comprehensive species descriptions are needed to further investigate the diversity of *Mercuria* species and their habitat types in the Mediterranean and Atlantic regions.

## Supplementary Material

XML Treatment for Mercuria bakeri

XML Treatment for Mercuria tingitana

XML Treatment for Mercuria targouasensis

XML Treatment for Mercuria midarensis

XML Treatment for Mercuria tensiftensis

## Supplementary materials

Supplementary material 1 Table 1–6 Data type: measurement Explanation note: Measurements recorded for the shell, radula, ctenidium, osphradium, digestive system, female and male genitalia, and nervous system in the *Mercuria* species examined. This is an open access article distributed under the terms of the Creative Commons Attribution License (CC BY 4.0), which permits unrestricted use, distribution, and reproduction in any medium, provided the original author and source are credited.Khadija Boulaassafer, Mohamed Ghamizi, Diana Delicado

## References

[B1] Akaike H (1974) A new look at the statistical model identification. IEEE Transactions on Automatic Control 19: 716–723. 10.1109/TAC.1974.1100705

[B2] Arconada B Ramos MA (2001) New data on Hydrobiidae systematics: two new genera from the Iberian Peninsula. Journal of Natural History 35: 949–984. 10.1080/002229301300323884

[B3] Ashby B Gupta S (2014) Parasitic castration promotes coevolutionary cycling but also imposes a cost on sex. Evolution 68: 2234–2244. 10.1111/evo.12425 24749747

[B4] BackHuys W Boeters HD (1974) Zur Kenntnis marokkanischer Binnenmollusken, I. Archiv für Molluskenkunde 104: 107–114.

[B5] Berrahou A Cellot B Richoux P (2001) Distribution longitudinale des macroinvertébrés benthiques de la Moulouya et de ses principaux affluents (Maroc). Annales de Limnologie – International Journal of Limnology 37: 223–235. 10.1051/limn/2001020

[B6] Boeters HD (1988) Moitessieriidae und Hydrobiidae in Spanien und Portugal. Archiv für Molluskenkunde 118: 181–261.

[B7] Boeters HD Falkner G (2017) The genus *Mercuria* Boeters, 1971 in France (Gastropoda: Caenogastropoda: Hydrobiidae). West-European Hydrobiidae, Part 13. Zoosystema 39: 227–261. 10.5252/z2017n2a4

[B8] Boulal M Boulanouar M Ghamizi M Boutin C (2017) Qualité de l’eau et faune aquatique des puits dans la région de Tiznit (Anti-Atlas occidental, Maroc). Bulletin de la Société d’Histoire Naturelle de Toulouse 153: 25–41.

[B9] Combes C Cheng TC (1986) Control of biomedically important molluscs. Archives de l’Institut Pasteur d’Algérie. Institut Pasteur d’Algérie 55: 153–193. 3079509

[B10] Darriba D Taboada GL Doallo R Posada D (2012) jModelTest 2: more models, new heuristics and parallel computing. Nature Methods 9: 772–772. 10.1038/nmeth.2109 PMC4594756 22847109

[B11] Davis GM Kitikoon V Temcharoen P (1976) Monograph on “*Lithoglyphosis*” *aperta*, the snail of Mekong River Schistosomiasis. Malacologia 15: 241–287. 948206

[B12] Davis GM Chen C-E Wu C Kuang T-F Xing X-G Li L Liu W-J Yan Y-L (1992) The Pomatiopsidae of Hunan, China (Gastropoda: Rissoacea). Malacologia 34: 143–342.

[B13] Davis GM Guo YH Hoagland KE Chen PL Zheng LC Yang HM Chen DJ Zhou YF (1986) Anatomy and Systematics of Triculini (Prosobranchia: Pomatiopsidae: Triculinae), freshwater snails from Yunnan, China, with descriptions of new species. Proceedings of the Academy of Natural Sciences of Philadelphia 138: 466–575.

[B14] Davis GM Kuo Y-H Hoagland KE Chen P-L Yang H-M Chen D-J (1984) *Kunmingia*, a new genus of Triculinae (Gastropoda: Pomatiopsidae) from China: Phenetic and cladistic relationships. Proceedings of the Academy of Natural Sciences of Philadelphia 136: 165–193.

[B15] Delicado D Machordom A Ramos MA (2012) Underestimated diversity of hydrobiid snails. The case of Pseudamnicola (Corrosella) (Mollusca: Caenogastropoda: Hydrobiidae). Journal of Natural History 46: 25–89. 10.1080/00222933.2011.623358

[B16] Delicado D Machordom A Ramos MA (2013) Living on the mountains: Patterns and causes of diversification in the springsnail subgenus Pseudamnicola (Corrosella) (Mollusca: Caenogastropoda: Hydrobiidae). Molecular Phylogenetics and Evolution 68: 387–397. 10.1016/j.ympev.2013.04.022 23660110

[B17] Delicado D Machordom A Ramos MA (2015) Effects of habitat transition on the evolutionary patterns of the microgastropod genus *Pseudamnicola* (Mollusca, Hydrobiidae). Zoologica Scripta 44: 403–417. 10.1111/zsc.12104

[B18] Diehl E Jauker B Albrecht C Wilke T Wolters V (2018) GIEßEN: University Collections: Justus Liebig University Gießen. Zoological Collections of Germany. Natural History Collections. Springer, Cham, 373–381. 10.1007/978-3-319-44321-8_29

[B19] Felsenstein J (1985) Confidence limits on phylogenies: An approach using the bootstrap. Evolution 39: 783–791. 10.2307/2408678 28561359

[B20] Folmer O Black M Hoeh W Lutz R Vrijenhoek R (1994) DNA primers for amplification of mitochondrial cytochrome c oxidase subunit I from diverse metazoan invertebrates. Molecular marine biology and biotechnology 3: 294–299. 7881515

[B21] García N Cuttelod A Malak DA (2010) The status and distribution of freshwater biodiversity in Northern Africa. IUCN, 156 pp.

[B22] Gargominy O Prie V Bichain J-M Cucherat X Fontaine B (2011) Liste de référence annotée des mollusques continentaux de France. Annotated checklist of the continental molluscs from France. MalaCo 7: 307–382.

[B23] Ghamizi M Vala JC Bouka H (1997) Le genre *Pseudamnicola* au Maroc avec description de *Pseudamnicolapallaryi* n. sp. (Gastropoda: Hydrobiidae). Haliotis 26: 33–49.

[B24] Girardi H (2004) Anatomie et biométrie de *Mercuriasimilis* (Draparnaud, 1805), (Gastropoda: Hydrobiidae) du Languedoc Roussillon, France. Documents Malacologiques 4: 83–86.

[B25] Giusti F (1979) Notulae malacologicae, 24. Il genere *Mercuria* (Prosobranchia: Hydrobiidae) nell’Isola di Sardegna (Studi sulla malacofauna di Sardegna e Corsica, 4). Archiv für Molluskenkunde 110: 1–14.

[B26] Glöer P Boeters HD Walther F Peter G Hans DB Frank W (2015) Species of the genus *Mercuria* Boeters, 1971 (Caenogastropoda: Truncatelloidea: Hydrobiidae) from the European Mediterranean region, Morocco and Madeira, with descriptions of new species. Folia Malacologica 23: 279–291. 10.12657/folmal.023.024

[B27] Glöer P Bouzid S Boeters HD (2010) Revision of the genera *Pseudamnicola* Paulucci 1878 and *Mercuria* Boeters 1971 from Algeria with particular emphasis on museum collections (Gastropoda: Prosobranchia: Hydrobiidae). Archiv für Molluskenkunde 139: 1–22. 10.1127/arch.moll/1869-0963/139/001-022

[B28] Guindon S Dufayard J-F Lefort V Anisimova M Hordijk W Gascuel O (2010) New algorithms and methods to estimate Maximum-Likelihood phylogenies: Assessing the performance of PhyML 3.0. Systematic Biology 59: 307–321. 10.1093/sysbio/syq010 20525638

[B29] Hasegawa M Kishino H Yano T (1985) Dating of the human-ape splitting by a molecular clock of mitochondrial DNA. Journal of Molecular Evolution 22: 160–174. 10.1007/BF02101694 3934395

[B30] Hershler R Liu H-P Bradford C (2013) Systematics of a widely distributed western North American springsnail, *Pyrgulopsismicrococcus* (Caenogastropoda, Hydrobiidae), with descriptions of three new congeners. ZooKeys 330: 27–52. 10.3897/zookeys.330.5852 PMC3800804 24146554

[B31] Hershler R Liu H-P Gustafson DL (2008) A second species of *Pyrgulopsis* (Hydrobiidae) from the Missouri River basin, with molecular evidence supporting faunal origin through Pliocene stream capture across the northern continental divide. Journal of Molluscan Studies 74(4): 403–413. 10.1093/mollus/eyn028

[B32] Hershler R Ponder WF (1998) A review of morphological characters of hydrobioid snails. Smithsonian Contributions to Zoology 600: 1–55. 10.5479/si.00810282.600

[B33] Hershler R Liu H-P Thompson FG (2003) Phylogenetic relationships of North American nymphophiline gastropods based on mitochondrial DNA sequences. Zoologica Scripta 32: 357–366. 10.1046/j.1463-6409.2003.00115.x

[B34] Holyoak DT Holyoak GA Mendes RMC (2017) Distribution and ecology of *Mercuriatachoensis* (Gastropoda: Hydrobiidae) in Portugal and evidence that *M.edmundi* is conspecific. Iberus 35: 203–210.

[B35] Huelsenbeck JP Ronquist F (2001) MRBAYES: Bayesian inference of phylogenetic trees. Bioinformatics (Oxford, England) 17: 754–755. 10.1093/bioinformatics/17.8.754 11524383

[B36] Hurvich CM Tsai C-L (1989) Regression and time series model selection in small samples. Biometrika 76: 297–307. 10.1093/biomet/76.2.297

[B37] Kadolsky D (2011) Nomenclatural comments on non-marine molluscs occurring in the British Isles. Journal of Conchology 41: 65–90.

[B38] Kistner EJ Dybdahl MF (2014) Parallel variation among populations in the shell morphology between sympatric native and invasive aquatic snails. Biological Invasions 16: 2615–2626. 10.1007/s10530-014-0691-4

[B39] Kumar S Stecher G Tamura K (2016) MEGA7: Molecular Evolutionary Genetics Analysis version 7.0 for bigger datasets. Molecular Biology and Evolution 33: 1870–1874. 10.1093/molbev/msw054 27004904 PMC8210823

[B40] Lim H-K Heyneman D (1972) Intramolluscan inter-trematode antagonism: a review of factors influencing the host-parasite system and its possible role in biological control. In: Dawes B (Ed.) Advances in Parasitology.Academic Press, 191–268. 10.1016/S0065-308X(08)60175-X 4559144

[B41] Mouahid A Mone H (1990) Interference of *Echinoparyphiumelegans* with the host-parasite system *Bulinustruncatus* – *Schistosomabovis* in natural conditions. Annals of Tropical Medicine & Parasitology 84: 341–348. 10.1080/00034983.1990.11812478 2260898

[B42] Paladilhe A (1866) Nouvelles miscellanées malacologiques. Chez Savy, Paris, 172 pp 10.5962/bhl.title.14440

[B43] Patzner RA Glöer P (2013) Süßwassermollusken von Ibiza (Balearen, Spanien). Linzer Biologische Beiträge 45: 837–844.

[B44] Puillandre N Lambert A Brouillet S Achaz G (2012) ABGD, Automatic Barcode Gap Discovery for primary species delimitation. Molecular Ecology 21: 1864–1877. 10.1111/j.1365-294X.2011.05239.x 21883587

[B45] Rambaut A (2010) FigTree. http:// tree.bio.ed.ac.uk/software/figtree

[B46] Rambaut A Suchard MA Xie D Drummond A (2014) Tracer v. 1.6. http://tree.bio.ed.ac.uk/software/tracer/

[B47] Ramos MA Arconada B Moreno D Rolán E (2000) A new genus and a new species of Hydrobiid snail (Mollusca: Gastropoda: Hydrobiidae) from eastern Spain. Malacologia 42: 75–101.

[B48] Ronquist F Huelsenbeck JP (2003) MrBayes 3: Bayesian phylogenetic inference under mixed models. Bioinformatics (Oxford, England) 19: 1572–1574. 10.1093/bioinformatics/btg180 12912839

[B49] Schreiber K Hauffe T Albrecht C Wilke T (2012) The role of barriers and gradients in differentiation processes of pyrgulinid microgastropods of Lake Ohrid. Hydrobiologia 682: 61–73. 10.1007/s10750-011-0864-4

[B50] Sugiura N (1978) Further analysts of the data by Akaike’ s information criterion and the finite corrections. Communications in Statistics – Theory and Methods 7: 13–26. 10.1080/03610927808827599

[B51] Taybi AF Mabrouki Y Ghamizi M Ali B (2017) The freshwater malacological composition of Moulouya’s watershed and Oriental Morocco. Journal of Materials and Environmental Sciences 8: 1401–1416.

[B52] Touabay M Aouad N Mathieu J (2002) Etude hydrobiologique d’un cours d’eau du Moyen-Atlas: l’oued Tizguit (Maroc). Annales de Limnologie 38: 65–80.

[B53] Urdy S Goudemand N Bucher H Chirat R (2010) Growth-dependent phenotypic variation of molluscan shells: Implications for allometric data interpretation. Journal of Experimental Zoology (Mol. Dev. Evol.) 314B: 303–326. 10.1002/jez.b.21338 20084667

[B54] Verhaegen G McElroy KE Bankers L Neiman M Haase M (2018) Adaptive phenotypic plasticity in a clonal invader. Ecology and Evolution 8: 4465–4483. 10.1002/ece3.4009 29760888 PMC5938463

[B55] Wilke T Davis GM (2000) Infraspecific mitochondrial sequence diversity in *Hydrobiaulvae* and *Hydrobiaventrosa* (Hydrobiidae: Rissooidea: Gastropoda): Do their different life histories affect biogeographic patterns and gene flow? Biological Journal of the Linnean Society 70: 89–105. 10.1111/j.1095-8312.2000.tb00202.x

[B56] Wilke T Davis GM Falniowski A Giusti F Bodon M Szarowska M (2001) Molecular systematics of Hydrobiidae (Mollusca: Gastropoda: Rissooidea): testing monophyly and phylogenetic relationships. Proc. Acad. Nat. Sci. Philadelphia 151(1): 1–21.

[B57] Wilke T Davis GM Qiu DC Spear RC (2006) Extreme mitochondrial sequence diversity in the intermediate schistosomiasis host *Oncomelaniahupensisrobertsoni*: another case of ancestral polymorphism? Malacologia 48: 143–157. 10.1635/0097-3157(2001)151[0001:MSOHMG]2.0.CO;2

[B58] Wilke T Rolán E Davis GM (2000) The mudsnail genus *Hydrobia* s.s. in the northern Atlantic and western Mediterranean: a phylogenetic hypothesis. Marine Biology 137: 827–833. 10.1007/s002270000407

[B59] Wilke T Haase M Hershler R Liu H-P Misof B Ponder W (2013) Pushing short DNA fragments to the limit: Phylogenetic relationships of “hydrobioid” gastropods (Caenogastropoda: Rissooidea). Molecular Phylogenetics and Evolution 66: 715–736. 10.1016/j.ympev.2012.10.025 23142112

