# Supplementary material for: The genus Mercuria Boeters, 1971 in Morocco: first molecular phylogeny of the genus and description of two new species (Caenogastropoda, Truncatelloidea, Hydrobiidae)
Source: Zookeys. 2018 Aug 27;(782):95–128. doi: 10.3897/zookeys.782.26797 (PMC6160864; doi:10.3897/zookeys.782.26797)
Supplement: Supplementary material 1 — Table 1–6 [file zookeys-782-095-s001.pdf]

Supplementary file 1

Table 1: Shell measurements (in mm) recorded in the Moroccan *Mercuria* species: *M. tensiftensis* sp. n. from 1, a ditch in Sidi Bouzid and 2, a pond near Lahjar Spring, Essaouira; 3, *M. midarensis* sp. n. from a ditch near Midar; *M. targouasensis* from 4, a ditch in Mirleft and 5, Oum Rbii Springs, Khenifra; 6, *M. tingitana* from a swampy area between Tangier and Ksar es Seghir.

	**1**	**2**	**3**	**4**	**5**	**6**
	Mean ± SD; CV (Max-Min) (n=18)	Mean ± SD; CV (Max-Min) (n=16)	Mean ± SD; CV (Max-Min) (n=29)	Mean ± SD; CV (Max-Min) (n=19)	Mean ± SD; CV (Max-Min) (n=19)	Mean ± SD; CV (Max-Min) (n=6)
SL	4.07±0.43; 0.11 (5.10–3.50)	3.50±0.22; 0.06 (3.95–3.06)	3.50±0.20; 0.06 (3.94–3.01)	2.90±0.29; 0.10 (3.43–2.63)	3.82±0.26; 0.06 (4.37–3.35)	3.31±0.29; 0.08 (3.60–2.93)
SW	2.75±0.25; 0.09 (3.23–2.33)	2.43±0.22; 0.09 (3.03–2.18)	2.42±0.15; 0.07 (2.71–2.07)	2.09±0.23; 0.11 (2.51–1.75)	2.86±0.27;0.09 (3.52–2.39)	2.11±0.17; 0.08 (2.27–1.88)
LBW	3.02±0.24; 0.08 (3.64–2.71)	2.74±0.20; 0.08 (3.22–2.41)	2.77±0.14; 0.05 (3.02–2.43)	2.32±0.21; 0.09 (2.71–2.06)	3.09±0.18; 0.05 (3.52–2.81)	2.49±0.27; 0.10 (2.74–2.09)
WBW	2.36±0.19;0.08 (2.74–2.12)	2.18±0.15; 0.07 (2.55–1.98)	2.17±0.12; 0.06 (2.45–1.81)	1.97±0.19; 0.10 (2.30–1.69)	2.32±0.11; 0.04 (2.69–2.21)	1.90±0.12; 0.06 (2.03–1.75)
SL–LBW	1.03±0.21;0.21 (1.46–0.69)	0.74±0.08; 0.11 (0.87–0.62)	0.72±0.11; 0.16 (0.97–0.55)	0.57±0.10; 0.18 (0.71–0.43)	0.71±0.12; 0.17 (0.94–0.44)	0.81±0.08; 0.10 (0.93–0.69)
AL	1.96±0.09; 0.05 (2.20–1.80)	1.73±0.14; 0.08 (2.11–1.56)	1.76±0.10; 0.06 (2.02–1.55)	1.50±0.16;0.11 (1.77–1.30)	1.98±0.12; 0.06 (2.26–1.78)	1.49±0.13; 0.09 (1.68–1.30)
AW	1.43±0.10; 0.07 (1.61–1.25)	1.33±0.12; 0.09 (1.56–1.15)	1.28±0.08; 0.06 (1.45–1.11)	1.10±0.13; 0.12 (1.26–0.81)	1.56±0.11; 0.07 (1.80–1.39)	1.08±0.12; 0.11 (1.20–0.92)
AH	1.96±0.11; 0.06 (2.22–1.79)	1.74±0.16; 0.09 (2.11–1.51)	1.80±0.09; 0.05 (2.02–1.65)	1.42±0.10; 0.08 (1.60–1.28)	2.06±0.17; 0.08 (2.53–1.85)	1.57±0.13; 0.08 (1.68–1.38)
WPW	1.44±0.24; 0.17 (1.92–1.10)	1.12±0.16; 0.14 (1.41–0.68)	1.19±0.09; 0.08 (1.7–0.7)	0.16±0.12; 0.11 (1.34–1.00)	1.26±0.11; 0.09 (1.50–0.99)	1.17±0.08; 0.07 (1.28–1.04)
WAW	0.23±0.08; 0.35 (0.41–0.12)	0.26±0.11; 0.41 (0.52–0.12)	0.22±0.07; 0.32 (0.35–0.06)	0.37±0.13; 0.37 (0.57–0.17)	0.23±0.09; 0.39 (0.45–0.09)	0.32±0.04; 0.14 (0.39–0.26)
NSW	4.44±0.51; 0.12 (5.00–4.00)	4.15±0.49; 0.12 (5.00–3.00)	4.16±0.36; 0.09 (5.00–4.00)	3.44±0.53; 0.15 (4.00–3.00)	4.11±0.43; 0.10 (5.00–3.00)	4.00±0; 0 (4.00–4.00)
SL/SW	1.47±0.09; 0.06 (1.66–1.35)	1.44±0.07; 0.05 (1.55–1.30)	1.41±0.16; 0.12 (1.58–0.85)	1.39±0.07; 0.05 (1.50–1.28)	1.34±0.09; 0.07 (1.58–1.21)	1.56±0.04; 0.03 (1.66–1.51)

Table 2: Radula formulae and measurements (in µm) recorded in the Moroccan *Mercuria* species: *M. tensiftensis* sp. n. from 1, a ditch in Sidi Bouzid and 2, a pond near Lahjar Spring, Essaouira; 3, *M. midarensis* sp. n. from a ditch near Midar; *M. targouasensis* from 4, a ditch in Mirleft and 5, Oum Rbii Springs, Khenifra; 6, *M. tingitana* from a swampy area between Tangier and Ksar es Seghir; 7, *M. bakeri* from a spring at 3.5 km N of Taghramt.

	**1**	**2**	**3**	**4**	**5**	**6**	**7**
Central teeth formula	4–C–4/1–1	(5)4–C–4(5)/1–1	(3)4–C–4(3)/1–1	3–C–3/1–1	(4)3–C–3(4)/1–1	4–C–4/1–1	(3)4–C–4(3)/1–1
Central teeth width	~ 20 µm	~ 20 µm	~ 20 µm	~ 20 µm	~ 30 µm	~ 20 µm	~ 15 µm
Lateral teeth formula	3–C–3	4–C–4	(2)3–C–3(2)	(4)3–C–3(4)	3–C–3	3–C–3	3–C–3
Cusps of inner marginal teeth	≥ 13	≥ 13	≥ 12	≥ 11	≥ 13	≥ 15	≥ 15
Cusps of outer marginal teeth	≥ 15	≥ 15	≥ 18	≥ 14	≥ 17	≥ 21	≥ 20
Radula length	~ 960µm	~ 800 µm	~ 850 µm	~ 750 µm	~ 960 µm	~ 600 µm	~ 750 µm
Radula width	~ 127µm	~ 100 µm	~ 120 µm	~ 100 µm	~ 150 µm	~ 100 µm	~ 100 µm
Number of rows	~ 60	~ 60	~ 55	~ 50	~ 55	~ 50	~ 60

Table 3: Ctenidium, osphradium and stomach measurements (in mm) recorded in the Moroccan *Mercuria* species: *M. tensiftensis* sp. n. from 1, a ditch in Sidi Bouzid and 2, a pond near Lahjar Spring, Essaouira; 3, *M. midarensis* sp. n. from a ditch near Midar; *M. targouasensis* from 4, a ditch in Mirleft and 5, from Oum Rbii Springs, Khenifra; 6, *M. tingitana* from a swampy area between Tangier and Ksar es Seghir; 7, *M. bakeri* from a spring at 3.5 km N of Taghramt. ND= No data

	**1** Mean ± SD; CV (Max-Min) (n=6)	**2** Mean ± SD; CV (Max-Min) (n=8)	**3** Mean ± SD; CV (Max-Min) (n=6)	**4** Mean ± SD; CV (Max-Min) (n=11)	**5** Mean ± SD; CV (Max-Min) (n=8)	**6** Mean ± SD; CV (Max-Min) (n=2)	**7** Mean ± SD; CV (Max-Min) (n=1)
CL	1.60±0.22; 0.14 (1.88–1.35)	1.19±0.26; 0.22 (1.49–0.85)	1.16±0.07; 0.06 (1.27–1.07)	0.95±0.14; 0.15 (1.22–0.76)	1.43±0.28; 0.20 (1.93–1.23)	1.23±0.21; 0.18 (1.38–1.08)	1.10
Os L	0.42±0.17; 0.40 (0.56–0.23)	0.42±0.09; 0.24 (0.54–0.27)	0.55±0.12; 0.22 (0.69–0.38)	0.34±0.09; 0.28 (0.59–0.26)	0.41±0.11; 0.28 (0.59–0.31)	0.47±0.04; 0.10 (0.50–0.43)	0.32
Os W	0.10±0.01; 0.17 (0.12–0.08)	0.10±0.01; 0.16 (0.12–0.08)	0.10±0.01; 0.16 (0.12–0.08)	0.09±0.01; 0.18 (0.12–0.06)	0.09±0.04; 0.42 (0.15–0.05)	0.11±0.01; 0.15 (0.12–0.09)	0.09
St L	1.13±0.17; 0.16 (1.40–0.88)	0.76±0.11; 0.15 (0.96–0.61)	1.03±0.11; 0.11 (1.16–0.84)	0.71±0.12; 0.18 (0.89–0.52)	0.94±0.05; 0.06 (1.03–0.87)	0.77	ND
St W	1.09±0.19; 0.18 (1.31–0.78)	0.79±0.15; 0.19 (0.99–0.55)	0.84±0.10; 0.12 (1.01–0.75)	0.75±0.11; 0.15 (0.96–0.57)	1.03±0.09; 0.09 (1.15–0.93)	0.82	ND
Ss L	0.97±0.15; 0.16 (1.26–0.82)	0.68±0.12; 0.18 (0.81–0.43)	0.68±0.07; 0.10 (0.74–0.56)	0.58±0.09; 0.16 (0.74–0.47)	0.93±0.16; 0.18 (1.21–0.77)	0.58	ND
Ss W	0.73±0.07; 0.10 (0.84–0.66)	0.43±0.06; 0.16 (0.55–0.34)	0.53±0.04; 0.08 (0.59–0.48)	0.46±0.06; 0.14 (0.55–0.32)	0.66±0.09; 0.14 (0.81–0.53)	0.47	ND

Table 4: Female genitalia measurements (in mm) recorded in the Moroccan *Mercuria* species: *M. tensiftensis* sp. n. from 1, a ditch in Sidi Bouzid and 2, from a pond near Lahjar Spring, Essaouira; 3, *M. midarensis* sp. n. from a ditch near Midar; *M. targouasensis* from 4, a ditch in Mirleft and 5, from Oum Rbii Springs, Khenifra; 6, *M. tingitana* from a swampy area between Tangier and Ksar es Seghir. ND= No data

	**1**	**2**	**3**	**4**	**5**	**6**
	Mean ± SD; CV (Max-Min) (n=4)	Mean ± SD; CV (Max-Min) (n=2)	Mean ± SD; CV (Max-Min) (n=4)	Mean ± SD; CV (Max-Min) (n=5)	Mean ± SD; CV (Max-Min) (n=8)	Mean ± SD; CV (Max-Min) (n=1)
Po L	2.53±0.62; 0.25 (3.41–1.92)	1.65±0.40; 0.24 (1.94–1.37)	1.89±0.23; 0.13 (2.13–1.56)	1.75±0.37; 0.22 (2.24–0.19)	2.18±0.44; 0.21 (3.12–1.71)	1.74
Po W	0.86±0.34; 0.40 (1.35–0.55)	0.49±0.07; 0.14 (0.54–0.44)	0.73±0.07; 0.10 (0.78–0.61)	0.57±0.09; 0.17 (0.68–0.42)	0.78±0.13; 0.17 (1.03–0.63)	0.63
Ag L	1.24±0.50; 0.40 (1.97–0.80)	0.68±0.46; 0.67 (1.03–0.36)	0.92±0.20; 0.22 (1.11–0.63)	0.77±0.17; 0.22 (0.98–0.51)	1.06±0.15; 0.14 (1.27–0.88)	0.68
Cg L	1.19±0.19; 0.16 (1.42–1.01)	0.83±0.23; 0.28 (1.00–0.67)	1.12±0.11; 0.10 (1.22–0.96)	0.81±0.15; 0.19 (0.98–0.59)	1.20±0.27; 0.23 (1.74–0.86)	0.80
SR1 L	0.20	0.21	0.21	0.12±0.02; 0.24 (0.16–0.09)	0.16±0.03; 0.21 (0.20–0.13)	0.22
BC L	1.09±0.23; 0.21 (1.23–0.74)	0.88±0.35; 0.40 (1.13–0.63)	1.00±0.14; 0.15 (1.20–0.88)	0.81±0.32; 0.29 (1.07–0.46)	1.09±0.23; 0.21 (1.59–0.88)	0.60
BC W	0.43±0.16; 0.37 (0.67–0.31)	0.35±0.17; 0.50 (0.47–0.23)	0.42±0.11; 0.28 (0.56–0.28)	0.33±0.12; 0.38 (0.47–0.17)	0.51±0.11; 0.23 (0.77–0.40)	0.39
dBC L	N.D.	0.23	N.D.	0.05	0.09	N.D.

Table 5: Male genitalia measurements (in mm) recorded in the Moroccan *Mercuria* species: *M. tensiftensis* sp. n. from 1, a ditch in Sidi Bouzid and 2, from a pond in Lahjar Spring, Essaouira; 3, *M. midarensis* sp. n. from a ditch near Midar; *M. targouasensis* from 4, a ditch in Mirleft and 5, from Oum Rbii Springs, Khenifra; 6, *M. bakeri* from a spring at 3.5 km N of Taghramt. ND= No data.

	**1**<br>Mean ± SD; CV<br>(Max-Min)<br>(n=2)	**2**<br>Mean ± SD; CV<br>(Max-Min)<br>(n=7)	**3**<br>Mean ± SD; CV<br>(Max-Min)<br>(n=4)	**4**<br>Mean ± SD; CV<br>(Max-Min)<br>(n=7)	**5**<br>Mean ± SD; CV<br>(Max-Min)<br>(n=2)	**6**<br>(n=1)
Pr L	1.80±0.13; 0.07<br>(1.89–1.71)	1.09±0.29; 0.27<br>(1.40–0.63)	0.99±0.07; 0.08<br>(1.10–0.92)	0.91±0.24; 0.27<br>(1.32–0.55)	1.11±0.29; 0.27<br>(1.32–0.90)	N.D.
Pr W	0.81±0.17; 0.22<br>(0.94–0.69)	0.47±0.11; 0.24<br>(0.61–0.28)	0.52±0.10; 0.20<br>(0.66–0.44)	0.35±0.11; 0.32<br>(0.55–0.21)	0.57±0.16; 0.28<br>(0.68–0.46)	N.D.
P L	1.34±0.79; 0.59<br>(1.90–0.77)	2.03±0.25; 0.12<br>(2.26–1.51)	2.98±0.36; 0.12<br>(3.25–2.57)	1.04±0.35; 0.34<br>(1.66–0.57)	2.46±0.11; 0.05<br>(2.54–2.38)	2.09
P W	0.38±0.19; 0.50<br>(0.52–0.24)	0.33±0.07; 0.23<br>(0.44–0.24)	0.46±0.09; 0.21<br>(0.57–0.39)	0.23±0.07; 0.31<br>(0.32–0.10)	0.46±0.11; 0.26<br>(0.54–0.37)	0.35
PA L	0.64±0.46; 0.71<br>(0.97–0.32)	0.55±0.15; 0.28<br>(0.69–0.32)	0.99±0.19; 0.20<br>(1.12–0.77)	0.57±0.19; 0.34<br>(0.78–0.28)	1.07±0.21; 0.20<br>(1.22–0.92)	0.60
PA W	0.39±0.21; 0.55<br>(0.54–0.23)	0.35±0.07; 0.20<br>(0.46–0.27)	0.49±0.07; 0.15<br>(0.55–0.44)	0.31±0.07; 0.22<br>(0.39–0.19)	0.65±0.11; 0.18<br>(0.73–0.56)	0.28
HeadL	1.31±0.25; 0.19<br>(1.49–1.13)	0.96±0.10; 0.11<br>(1.17–0.85)	1.14±0.16; 0.14<br>(1.32–1.01)	0.84±0.09; 0.11<br>(0.97–0.71)	1.41±0.05; 0.05<br>(1.45–1.37)	0.85
PL/PA L	2.19±0.33; 0.15<br>(2.42–1.96)	3.87±0.90; 0.23<br>(5.24–2.96)	3.03±0.27; 0.09<br>(3.33–2.85)	1.91±0.56; 0.29<br>(2.96–1.33)	2.35±0.57; 0.24<br>(2.75–1.94)	3.46
PL/Head L	0.98±0.42; 0.43<br>(1.28–0.69)	2.11±0.27; 0.13<br>(2.44–1.77)	2.61±0.29; 0.11<br>(2.92–2.35)	1.25±0.46; 0.37<br>(2.15–0.62)	1.74±0.01; 0.01<br>(1.75–1.73)	2.45
PA L/HeadL	0.47±0.26; 0.56<br>(0.65–0.28)	0.57±0.14; 0.25<br>(0.75–0.37)	0.87±0.13; 0.15<br>(1.01–0.76)	0.69±0.25; 0.36<br>(0.96–0.36)	0.76±0.18; 0.24<br>(0.89–0.63)	0.71

Table 6: Nervous system measurements (in mm) recorded in the Moroccan *Mercuria* species: *M. tensiftensis* sp. n. from 1, a ditch in Sidi Bouzid and 2, from a pond near Lahjar Spring, Essaouira; 3, *M. midarensis* sp. n. from a ditch near Midar; *M. targouasensis* from 4, a ditch in Mirleft and 5, from Oum Rbii Springs, Khenifra. ND= No data.

	**1**	**2**	**3**	**4**	**5**
	Mean ± SD; CV (Max-Min) (n=3)	Mean ± SD; CV (Max-Min) (n=5)	Mean ± SD; CV (Max-Min) (n=4)	Mean ± SD; CV (Max-Min) (n=1)	Mean ± SD; CV (Max-Min) (n=3)
LRCG	0.26±0.02; 0.09 (0.29–0.24)	0.22±0.03; 0.16 (0.26–0.18)	0.25±0.06; 0.25 (0.33–0.17)	0.29	0.28±0.04; 0.17 (0.31–0.22)
LLCG	0.26±0.02; 0.08 (0.28–0.24)	0.21±0.02; 0.10 (0.24–0.19)	0.26±0.09; 0.37 (0.37–0.14)	0.27	0.25±0.05; 0.22 (0.31–0.21)
LCC	0.09±0.02; 0.25 (0.12–0.07)	0.12±0.01; 0.12 (0.14–0.10)	0.15±0.06; 0.44 (0.25–0.09)	0.13	0.13±0.004; 0.03 (0.14–0.13)
LRPG	0.12±0.02; 0.24 (0.14–0.10)	0.09±0.01; 0.16 (0.10–0.08)	0.09±0.03; 0.32 (0.11–0.07)	0.14	0.10
LLPG	N.D.	0.12±0.01; 0.08 (0.14–0.12)	0.10±0.03; 0.29 (0.13–0.06)	0.17	0.15±0.01; 0.08 (0.16–0.14)
LPsupC	0.36±0.09; 0.27 (0.43–0.25)	0.33±0.05; 0.16 (0.39–0.25)	0.47±0.15; 0.32 (0.69–0.38)	0.56	0.54±0.15; 0.29 (0.69–0.38)
LPsubC	N.D.	0.07±0.01; 0.16 (0.08–0.06)	0.013	N.D.	0.02±0.01; 0.58 (0.03–0.01)
LsupG	0.12±0.07; 0.05 (0.13–0.12)	0.07±0.01; 0.20 (0.08–0.05)	0.16±0.08; 0.52 (0.29–0.09)	0.09	0.12±0.01; 0.05 (0.13–0.12)
LsubG	N.D.	0.09±0.008; 0.09 (0.10–0.09)	0.10±0.01; 0.14 (0.12–0.09)	0.06	0.11±0.02; 0.24 (0.14–0.08)
RPG	0.64±0.02; 0.03 (0.65–0.61)	0.79±0.14; 0.18 (1.00–0.64)	0.70±0.15; 0.22 (0.81–0.48)	0.70	0.76±0.12; 0.15 (0.84–0.62)
